# Advances in Materials for Soft Stretchable Conductors and Their Behavior under Mechanical Deformation

**DOI:** 10.3390/polym12071454

**Published:** 2020-06-29

**Authors:** Thao Nguyen, Michelle Khine

**Affiliations:** 1 Department of Chemical and Biomolecular Engineering, University of California, Irvine, CA 92697, USA; thaon8@uci.edu; 2 Department of Biomedical Engineering, University of California, Irvine, CA 92697, USA

**Keywords:** soft conductors, wearable technology, stretchable electronics

## Abstract

Soft stretchable sensors rely on polymers that not only withstand large deformations while retaining functionality but also allow for ease of application to couple with the body to capture subtle physiological signals. They have been applied towards motion detection and healthcare monitoring and can be integrated into multifunctional sensing platforms for enhanced human machine interface. Most advances in sensor development, however, have been aimed towards active materials where nearly all approaches rely on a silicone-based substrate for mechanical stability and stretchability. While silicone use has been advantageous in academic settings, conventional silicones cannot offer self-healing capability and can suffer from manufacturing limitations. This review aims to cover recent advances made in polymer materials for soft stretchable conductors. New developments in substrate materials that are compliant and stretchable but also contain self-healing properties and self-adhesive capabilities are desirable for the mechanical improvement of stretchable electronics. We focus on materials for stretchable conductors and explore how mechanical deformation impacts their performance, summarizing active and substrate materials, sensor performance criteria, and applications.

## 1. Introduction

**Figure 1:**
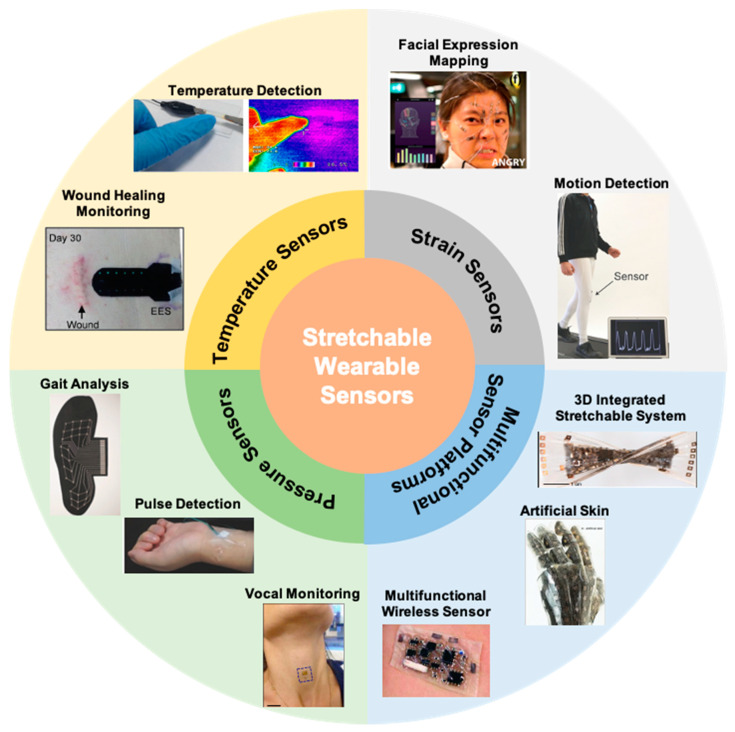
Illustration of recently developed wearable mechanical sensors. Temperature sensors: Wound Healing Monitoring [21]. Temperature Detection [22]. Strain Sensors: Facial Expression Mapping [4]. Motion Detection [23]. Multifunctional Sensor Platforms: 3D Integrated Stretchable System [24]. Artificial Skin [25]. Multifunctional Wireless Sensors [26]. Pressure Sensors: Vocal Monitoring [27]. Pulse Detection [28]. Gait Analysis [29]. Reproduced with permission from [21]. Copyright 2014, John Wiley and Sons. Reprinted with permission from [22]. Copyright 2018, American Chemical Society. Reprinted with permission from [4]. Copyright 2018, American Chemical Society https://pubs.acs.org/doi/10.1021/acsnano.8b05019. Further permissions related to the material excerpted should be directed to the AC. Reproduced with permission from [23]. Copyright 2015, John Wiley and Sons. Reproduced with permission from [24]. Copyright 2018, Spring Nature. Reproduced with permission from [25]. Copyright 2014, Springer Nature. Reproduced with permission [26]. Copyright 2014, The American Association for the Advancement of Science. Reproduced with permission from [27]. Copyright 2014, Springer Nature. Reproduced with permission from [28]. Copyright 2017, John Wiley and Sons. Reproduced with permission from [29]. Copyright 2017, John Wiley and Sons.

Wearable electronics have the ability to push the boundaries of human interaction with technology. Most are familiar with smart devices such as fitness monitors and smart watches that report on basic information such as heart rate or number of steps. These devices, however, still rely on conventional electronics that have rigid components. There is greater demand for components that can provide accurate, reliable data without impeding natural movement. Soft, stretchable sensors have gained much interest as they can withstand large deformations while retaining functionality and conformality to the body. Desirable characteristics include soft compliance for minimum discomfort, direct application to the skin, improved signal fidelity, quick response time, and ease of use. Wearable sensors have been applied towards motion detection [1] and rehabilitation [2] along with facial detection [3,4], demonstrated for potential health monitoring [5,6,7,8], and integrated into sensing platforms for human-machine interface [9,10] as seen in Figure 1. More recent research advances have turned towards introducing self-healing capabilities [11,12,13], optical transparency [3,14,15,16], and building multimodal functionality for more sophisticated devices [17,18,19,20].

As most conductive materials tend to be rigid, researchers have adopted a few common approaches to make these materials stretchable such as integrating deterministic geometrics (e.g., wrinkled, serpentine, cracked, or mesh structures) into active conductive materials for added strain relief or leveraging intrinsically stretchable conductive materials like liquid metals and conductive polymers. Other strategies involve creating composites by dispersing conductive fillers into a polymer matrix or introducing a hybrid combined structure that involves multiple conductive elements. Nearly all these approaches rely on a silicone-based elastomer as support to aid stretchability. While silicone elastomers support greater stretchability in more rigid active materials, inherent mechanical mismatches at the interface between the active material and the underlying substrate limit mechanical reliability. Conventional silicone elastomers also cannot offer self-healing abilities for enhanced robustness and lack strong adhesion for simple attachment to the human body. Moreover, standard printed circuit board (PCB) manufacturing processes are incompatible with silicone use, which has been shown to contaminate downstream processes with residue, even when the presence of silicone is not visible to the eye [30,31,32].

Specifically, silicones have low surface energy, allowing them to wet most surfaces readily, and may be easily transferred from process to process through poor housekeeping. Further, contaminates can impact silicone curing, leaving partially uncured residue, while silicone oils are also often added as softening agents but can escape the cured matrix. Silicone residue can easily migrate from surfaces, including onto manufacturing equipment, and spread in a near imperceptible film, causing adhesion failure in subsequent bonding steps (e.g., wire bonding) [30]. Cleaning methods with solvent may remove some of the residue, but bonding adhesion rarely ever returns entirely to baseline [32]. Properties of silicone substrates and other polymer materials are further outlined in Section 2. While most research focus has been aimed towards making rigid active materials more stretchable and leveraging commercially available stretchable polymer substrates as support, development of new polymer materials would allow for potential mechanical improvements in stretchable electronics. Engineering materials which are not only compliant and stretchable but also have self-healing capabilities and self-adhesive properties would be highly desirable. Stretchable electronics require both electrical and mechanical integrity in order to reach the stage of commercially available electronic devices. 

Here, we review recent advances in materials for creating stretchable soft conductors. This review focuses on material choice for stretchable electronics and how mechanical deformation impacts performance. Although many wearable reviews cover the mechanical design of active materials, and there are separate review papers devoted to self-healing electronics or biocompatible adhesives specifically, there are few comprehensive reviews that put those topics in context with other wearable devices in the same category. We aim to cover common substrate and functional materials, general stretchable sensor performance criteria, and applications. The next section covers polymer substrate materials and summarizes functional materials considerations. Intrinsically stretchable conductive materials such as ionic conductors, conductive polymers, and liquid metals will also be discussed in this section. Additionally, we outline sensor performance metrics and advanced functional properties in next generation soft stretchable electronics. Finally, current applications in motion detection and rehabilitation, healthcare monitoring, and consumer use will be discussed along with a brief outlook on the field of stretchable electronics. 

## 2. Materials Considerations 

As previously mentioned, there are a few common strategies to create stretchable electronics: adding strain relieving structures to conventional conductive materials, utilization of intrinsic stretchable conductors, or combined design of a composite material. There is strong research interest in skin-mountable wearable pressure, strain, and temperature sensors, and a wide range of materials have been utilized to design these types of sensors. The choice of substrate materials and intrinsically stretchable conductive materials are covered in this section. For completeness, a brief summary of traditional functional materials has also been provided in this section, but more than a few reviews cover this particular class of active materials research for stretchable electronics development in further detail [18,33,34,35,36,37,38]. 

### 2.1. Substrate Materials

For soft wearable sensors, the supporting material would ideally allow for great mechanical versatility, easy processing, and good adhesion to functional materials along with being biocompatible, chemically inert, and low cost. Potential support materials include polymer classes such as elastomers and hydrogels which are discussed in the subsequent sections.

#### 2.1.1. Elastomers

In particular, the elasticity of elastomers is a key aspect that allows stretchable electronics to withstand repetitive deformation without damage. Silicone elastomers are most widely used as they display high stretchability, simple curing processability, and have tunable mechanical properties. The most common silicone elastomers, polydimethylsiloxane (PDMS) (Sylgard-184) and Ecoflex (Smooth-On), are commercially available, biocompatible, and have elastic moduli ranges comparable to that of skin (30 kPa), as seen in Table 1 [35]. The properties of PDMS, in particular, have been well studied, and it has been widely used in soft lithography [39,40]. 

Non-silicone elastomers include thermoplastic elastomers such as polyurethane (PU or TPU for thermoplastic polyurethane) and block copolymers (i.e., SEBS) which are all physically crosslinked elastomers that also have high stretchability. Thermoplastic elastomers can be processed as thermoplastics, allowing them to be re-melted, extruded, or injection molded, unlike chemically crosslinked silicone elastomers. This ease of processability makes thermoplastic elastomers an especially attractive option for printing conductive inks. That being said, thermoplastic elastomers must have fabrication temperature below that of the hard phase (i.e., the styrene component) as decomposition occurs at high temperature (~200 °C). Further, block copolymers used as substrate materials for stretchable sensors have been largely limited to polystyrene-based elastomers, their viscoelastic properties have large impact on reliable electrical performance, and their compliance can also be several orders of magnitude higher than that of silicone elastomers or human skin [41].

Although the elastomeric material often acts as a non-conductive polymer support layer that interfaces with a separate active material layer, conductive fillers (e.g., nanoparticles, carbon nanotubes) can also be dispersed within the polymer matrix to create composite stretchable sensors [42,43,44]. Composite sensors, however, are often not as conductive as their bulk materials counterparts, and filler content can change the mechanical properties of the elastomer. The challenge lies in balancing the filler material and polymer matrix in order to promote both electron transport and mechanical compliance.

#### 2.1.2. Hydrogels

**Figure 2:**
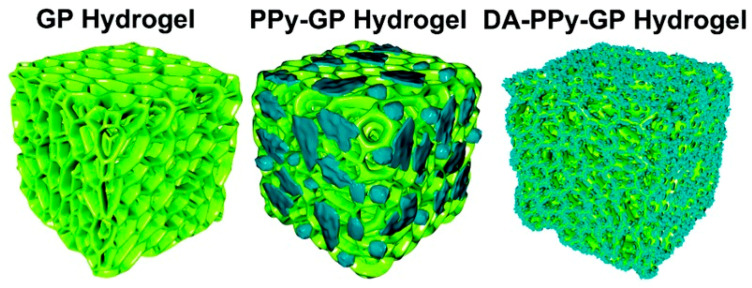
A schematic of structure and morphology for an elastomeric gelatin methacrylate-polyacrylamide (GP) double network hydrogel, polypyrrole (PPy) incorporated GP hydrogel, and dopamine (DA)-PPy-GP hydrogel. Reproduced with permission from [59]. Copyright 2019, Royal Society of Chemistry.

Hydrogels are a potential class of support material for soft wearable sensors as they are hydrophilic polymer networks that can closely resemble biological tissue due to their high water content and soft, rubbery consistency. Moreover, hydrogel materials are tunable, adaptable, stimuli-responsive, biocompatible, and have low interfacial tension with human tissue [45]. Given the physiological and mechanical resemblance to human tissue, hydrogels can offer ideal matrix components for soft stretchable electronics [46,47,48,49,50,51,52]. Common hydrogels, however, can suffer from low mechanical robustness and limited stretchability. The emergence of tough hydrogels has resulted in high mechanical strength, but the challenge remains to craft robust, stretchable, and biocompatible hydrogel matrixes for novel stretchable electronics. Tough hydrogel composition requires an elastic long chain polymer network along with a dissipative polymer network to allow for both stretchability and mechanical strength [53,54]. As with conventional elastomers, conductive filler material can also be incorporated into the matrix of hydrogels, but this blending method tends to require high filler content which disturbs the crosslinking and weakens the mechanical properties of conductive hydrogels. Innovations in polymer chemistry and composite formulations have led to in situ polymerization synthesis of hybrid hydrogels through the incorporation of graphene aerogels [55], modified silver nanowire aerogels [56], and conductive polymers [57,58,59,60] into the hydrogel scaffold to form stretchable conductors. An example of one such conductive polymer integrated into the hydrogel matrix can be seen in Figure 2.

Hydrogels are a versatile materials choice for stretchable electronics, but given their high water content, avoiding property changes upon evaporation remains difficult. Prevention of hydrogel dehydration involves adding hygroscopic salts or humectants to the hydrogel or encasing the hydrogel with a conventional elastomer [45]. Expanding upon the use of a conventional elastomer, Yuk et al. introduce a hydrogel-elastomer hybrid to prevent water evaporation. This method involves interpenetrating covalently crosslinked stretchy polymer networks and physically crosslinked dissipative networks to form a tough hydrogel before placing the hydrogel in contact with a benzophenone-treated elastomer and grafting the two materials together with ultraviolet light to form a hybrid structure [61]. Moreover, this method can also be applied to a number of conventional elastomers (Sylgard 184 PDMS, polyurethane, latex, Ecoflex) and tough hydrogels, including polyacrylamide (PAAm)-based and polyethylene glycol diacrylate (PEGDA)-based hydrogels. Achieving strong adhesion to other materials also remains a key challenge with hydrogels. A promising avenue involves silane functionalization of certain solid surfaces (glass, ceramic, metal) and covalently bonding the hydrogel’s polymer network to the solid surface through radical polymerization during hydrogel formation [62]. Along those lines, silane coupling agents can also be introduced into the precursor solutions of both the hydrogel and the elastomer, allowing the two materials to be grafted together [63]. Another approach applies cyanoacrylate/alkane solution as a bonding agent on substrate surfaces and presses the hydrogel onto the substrate to accelerate the polymerization process [64].

Moreover, as hydrogels contain a polymer matrix and water molecules, they can also be turned into an ionic conductor with the addition of ions or ionic salts. Details about ionically conductive hydrogels can be found under Section 2.3.2. In addition to their versatility, desired attributes in hydrogel-based—and other polymer materials—sensors are mechanical toughness, high conductivity, self-healing ability, and self-adhesive properties. The latter two attributes are discussed further in Section 3.6 and Section 3.7.

### 2.2. Traditional Functional Materials

**Figure 3:**
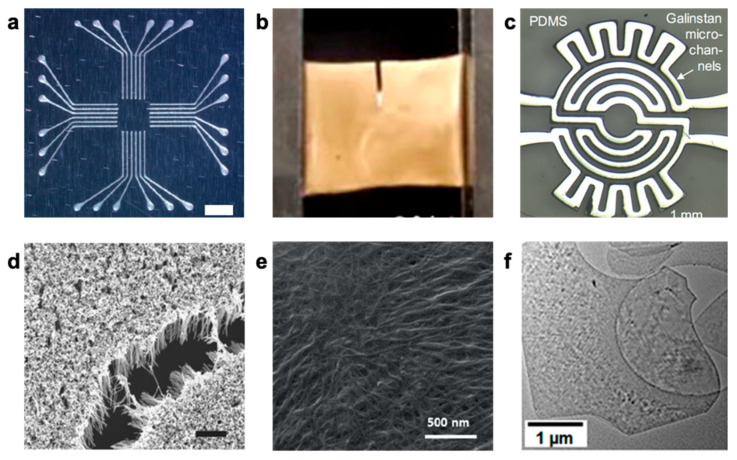
Various active materials for stretchable electronics. (**a**) Silver flake/polyurethane ink. (**b**) Gold thin film. (**c**) Liquid metal. (**d**) Gold nanowires. (**e**) Carbon nanotubes (CNTs). (**f**) MXene nanosheets. (**a**) Reprinted with permission from [29]. Copyright 2017, John Wiley and Sons. (**b**) Reprinted with permission from [65]. Copyright 2018, American Chemical Society. (**c**) Reproduced with permission from [66]. Copyright 2017, John Wiley and Sons. (**d**) Reproduced with permission from [67]. Copyright 2019, John Wiley and Sons. (**e**) Reprinted with permission from [68]. Copyright 2019, American Chemical Society. (**f**) Reproduced [69]. Adapted and reproduced with permission as licensed under the Creative Commons Attribution 4.0 International License.

There has been promising development in novel active materials and materials design in the past few decades. Advanced manufacturing has led to the rise of micro and nanoscale level features in bulk materials, allowing active material selection for soft stretchable electronics fabrication to be quite diverse. Selection ranges from conventional conductive elements (e.g., metallic or semiconductor thin films) to nanomaterials (e.g., carbon nanotubes (CNTs), nanowires and/or nanoparticles) and other 2D materials (e.g., graphene, MXene, and metallic nanosheets) (Figure 3). Other functional materials include conductive inks and liquid metals.

Metals are traditionally used to create conductive traces of a circuit due to their high electrical conductivity. They are often deposited as thin films (<1 μm thick) onto compliant substrates for stretchable electronics [70,71,72,73]. Gold, platinum, and silver films are widely used as electrodes that interface with skin as they have low contact resistance and are chemically inert [74]. Although planar metallic and semiconductor films can be made moderately stretchable with support of a polymer substrate (20–30% strain in comparison to <5% strain found in unsupported metal films) [70,75], they often still require engineering designs such as wavy, buckling or serpentine patterning for additional strain relief. Much of that work has been pioneered by Rogers’ group and adopted by many researchers since. Further details can be found in recent reviews on structural approaches to stretchable electronics [42,76,77]. Rogers’ group has also made great strides towards commercially viable devices with a combination of serpentine patterning and silicone elastomers; however, these devices are slightly less elegantly constructed than their academic counterparts [78,79]. Although there have been advances in commercialization, some compromises must be made to adjust for manufacturing.

Conductive nanomaterials have also emerged as a new class of active materials for stretchable electronic construction. In particular, silver nanowires (AgNWs) have proven popular for their high conductivity, large aspect ratio, and low percolation threshold requirements [14,18,80,81,82,83,84,85]. Moreover, they can be easily synthesized with tunable physical properties and can be solution processed with drop casting, vacuum filtration, and spray deposition; they have been largely studied for their high electrical performance and optical transparency [86]. While AgNWs are still subject to oxidation [87,88,89,90], other metal nanowires have even more rapid oxidation (e.g., copper nanowires [91,92]) or remain costly (i.e., gold nanowires (AuNWs) [4]), which can compromise stable conductivity. Methods of mitigating nanowire oxidation remain an active area of research [93,94]. Nanowire performance is dependent upon aspect ratio, loading density, and interfacial adhesion between the nanowires and the substrate. Nanoparticles are another emerging type of active material that can retain high conductivity and be suspended in solvent with good solvent stability. For example, silver flakes have been utilized for their versatility and printing compatibility [29,95]. However, they require large volume fraction for electrical percolation, have weak interaction with polymer matrices, and are prone to inhomogeneous distribution of the particles; these factors can compromise the mechanical properties of resulting composite material. 

Carbon-based nanomaterials (e.g., carbon black, CNTs, graphene, reduced graphene oxide, and carbon fibers) are another promising class of materials for their electrical conductivity, chemical stability, and mechanical strength [96,97,98,99,100,101,102,103,104]. Although carbon-based materials are less conductive than metals, they require low percolation thresholds in order retain electrical conductivity. Graphene is a two-dimensional material with excellent optical, electrical, and mechanical properties, but obtaining high quality graphene with large area and high stretchability still remains a major challenge to produce [105]. Future applications for graphene-based wearable sensors would require advancements in manufacturing to simplify fabrication and reduce the cost as synthesis of graphene still remains expensive, laborious, and difficult to scale [106].

### 2.3. Intrinsic Stretchable Functional Materials

Intrinsic stretchable conductors such as liquid metals, ionic conductors, and conductive polymers represent a new generation of wearable electronic materials. Chemical modifications also allow them to be designed with self-healing capabilities and self-adhesive properties in addition to conductivity. These advanced properties will be further discussed in Section 3.6 and Section 3.7. 

#### 2.3.1. Liquid Metals

**Figure 4:**
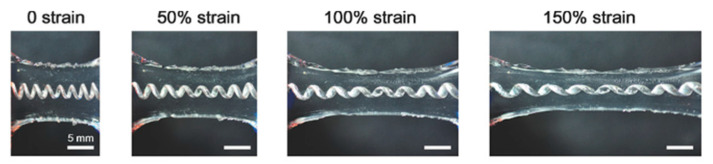
Demonstration of eutectic gallium indium (EGaIn) encased in polyacrylamide-alginate hydrogel undergoing strain. Reproduced with permission from [107]. Copyright 2018, John Wiley and Sons.

As liquid metals are liquid at room temperature, they can retain both metallic and fluidic properties. They exhibit excellent stretchability (as seen in Figure 4) [107] and electrical conductivity (3.4 × 10^4^ S cm^−1^) [108]. Mercury is one commonly known liquid metal that is toxic, making it an unsuitable choice as a wearable stretchable material. As such, much focus has turned to low toxicity liquid metals based on gallium such as eutectic gallium indium (EGaIn) and Galinstan (GaInSn). Liquid metals rapidly form a very thin oxide layer when exposed to oxygen under ambient conditions. This oxide layer helps the metal adhere to surfaces and gives liquid metals self-healing properties as reconnected liquid metal merges readily due to high surface tension but does not cause noticeable interference with the electron transfer at the interface [108]. In fact, the presence of an oxide layer aids in patterning. 

Researchers have also made recent strides in composite materials by studying liquid metal microdroplet formation in order to more precisely pattern stretchable electronics. For instance, Kim et al. explored EGaIn wettability on other conductors, demonstrating selective printing of liquid metals on honeycomb-structured gold nanosheets supported by PDMS [109]. Wang et al. anchored conductive fillers with EGaIn particles to produce printable and superelastic conductors [110]. Xu et al. cleverly disperse Nickel (Ni) micoparticles into EGaIn and deploy a permanent magnet to flow the liquid metal droplet through a shadow mask [111]. These Ni particles also aided adhesion between the liquid metal and the underlying hydrogel substrate. Jeong et al. initially deposit a gold thin film onto PDMS before casting a GaInSn droplet with native oxide layer [112]. Then, a few microliters of 10 wt% sodium hydroxide (NaOH) are cast onto the sample, reducing the liquid metal to selectively coat the gold surface. Liquid metal conductors can produce ultra-stretchable conductors (>500% strain) with compatible electromechanical coupling with a polymer matrix and show promise as stretchable interconnects [110,113,114,115]. 

#### 2.3.2. Ionic Conductors

Ionic conductors are often composed of hydrogels with ions or ionic liquids. Ionic hydrogels have potential as soft strain sensors as they have high compliance, stretchability and conductivity [116,117]. These materials maintain softness and have tunable mechanical elasticity with an elastic modulus ranging from 1 kPa to 100 kPa [118]. Also, ionic mobility—and, thus, ionic conductivity, is negligibly affected by strain. Moreover, ionic conductors exhibit excellent stretchability (>600%) and have high transparency [119,120]. They also have the ability to form electric double layers at the interface when paired with conventional electrical conductors [121]. The electric double layer operates like a capacitor where excess charge on the electrical conductor layer is compensated by an accumulation of oppositely charged excess ions in the ionic conductor. While this allows for the creation of electric double layer-based supercapacitors [122,123,124,125,126,127], the presence of an electric double layer also makes it difficult to operate with continuous direct current (DC) and would require alternate current (AC) operation. 

**Figure 5:**
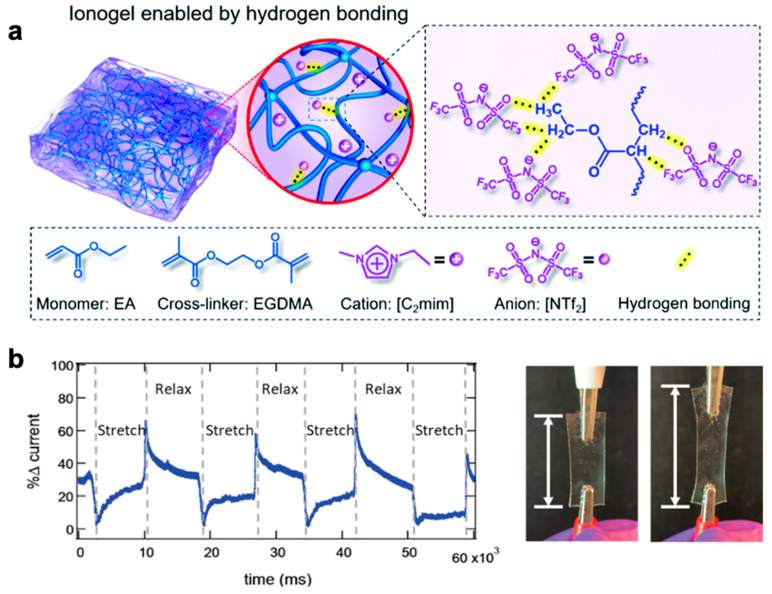
(**a**) Schematic description of a transparent, mechanically robust, and stable ionogel enabled by hydrogen bonding. Reproduced with permission from [128]. Copyright 2020, Royal Society of Chemistry (**b**) Mechanical characterization of a 3D printed crosslinked ionogel. Reproduced with permission from [129]. Copyright 2019, John Wiley and Sons.

Ionic conductors are not confined to hydrogels and can also be formed as ionogels—ionic liquid-based gel systems—and also elastomers that include ions or ionic liquids. Ionogels are a new class of soft materials with ionic conductivity and thermal stability, and unlike most hydrogels, do not dry out in open air, offering a promising option for soft stretchable conductors [13,120,128,129,130,131,132]. Figure 5 depicts examples of transparent ionogels (Figure 5a,b) along with a representative demonstration of mechanical strain (Figure 5b). As a relatively nascent category of materials, their ionic conductivity is often lower than that of conductive hydrogels—both ionic and with conductive fillers—the challenge remains to develop ionogels with high ionic conductivity, transparency, stretchability, and reliability. Shi et al. created ionic conducting elastomers that are synthesized by dissolving salt into the monomer prior to curing and achieves conductivity by ionic transport through the polymer chains, making it solvent free. This allows the ionic elastomer to remain stable in air without decay in stretchability, transparency, and conductivity [133]. Being solvent-free, this material would be noncorrosive to standard metal electrodes, giving it interfacial advantages with integration to electrical interconnects. Other ionic elastomers involve polymer synthesis of new ionic liquids such as deep eutectic solvents for stretchable electronics [134].

#### 2.3.3. Conductive Polymers

Conductive polymers are soft conductive materials that can offer tunability in molecular structure along with electrical and mechanical properties. However, there remains a challenge in maintaining both high conductivity and high stretchability. Often, high conductivity comes with high crystallinity and low insulating content, resulting in low stretchability. Poly(3,4-ethylenedioxythiophene):poly(styrenesulfonate) (PEDOT:PSS) is a promising conductive polymer with the highest reported conductivity among solution-processed polymers, but the semicrystalline nature of both PEDOT and PSS limits stretchability to ~5%. Potential solutions are to introduce a plasticizer or to use ionic salts. Ionic salts have been shown to even enhance the conductivity of PEDOT:PSS through morphological changes and doping. For instance, Wang et al. produce PEDOT:PSS films capable of stretching to 100% strain with a conductivity >4100 S cm^−1^ through the addition of ionic additives-assisted stretchability and electrical conductivity (STEC) enhancers. These STEC enhancers soften the PSS domains and provide better connectivity and crystallinity of the PEDOT regions along with enhanced electrical conductivity through doping [135]. Another technique to increase stretchability is to blend it with soft elastomers like PDMS [136] or polyurethane [137]. By blending PEDOT:PSS with PDMS, Noh et al. were able to extend stretchability to 75% strain while retaining comparable conductivity to pure PEDOT:PSS [138]. Hansen et al. are able to extend further stretchability to 200% strain with blending PEDOT:PSS with polyurethane with high conductivity to 50% (120 S cm^−1^) and lower conductivity past that strain point (30 S cm^−1^) [139]. 

Other approaches to improve mechanical properties involve processing conductive polymers as hydrogels through incorporation of another polymer network. Feig et al. were able to successfully synthesize PEDOT:PSS hydrogels with high stretchability (>100%) and conductivity (>0.1 S cm^−1^) through controlling the gelation to form conducting interpenetrating networks [140]. Moreover, this fabrication method requires low levels of PEDOT:PSS to form conductive connected pathways and maintain mechanical properties that are comparable to that of biological tissue. They report a conductivity of 0.23 S cm^−1^ which was a record for PEDOT:PSS hydrogels at the time (2018) with low PEDOT:PSS weight content. Other PEDOT:PSS hydrogels have been reported at significantly higher conductivities: Yao et al. reach 8.8 S cm^−1^ after concentrated sulfuric acid treatment [141] whereas Lu et al. display 40 S cm^−1^ with the addition of dimethyl sulfoxide and dry annealing application [142]. Liu et al. take it another step further with a reported conductivity of 47 S cm^−1^ by blending the conductive polymer with ionic liquids before removing the ionic liquid additive through water exchange. The ionic liquid itself does not electrically contribute to the final conductivity value but rather facilitates further removal of PSS and modify the PEDOT structure to allow for effective interconnected structures. The researchers attribute both effects to the high conductivity found in the resulting PEDOT:PSS hydrogel [143]. That said, it should be noted that these particular hydrogels with high conductivities have limited demonstrated stretchability to <20% strain.

While PEDOT:PSS is one of the more often explored conductive polymers, other types of conductive polymers include polyaniline (PANI) [144,145,146,147] and polypyrrole (PPy) [148,149,150] and conductive hydrogels blends like polydopamine/polyvinyl alcohol hydrogel (PDA/PVA) [151] through the incorporation of conductive polymers into a hydrogel matrix to create materials that are more mechanically compliant and comparable to soft biological tissue. Conductive polymers can also be blended into a thermoplastic elastomer such SEBS due to their high surface energy compatibility for improved mechanical properties [152,153,154,155,156].

## 3. Mechanical Sensor Characteristics of Interest

The materials described in the previous section are typical components for fabrication of stretchable electronics. There are many types of conductive elements (e.g., wires, sensors), and each will have different electrical properties. For example, material choice for a wire should have very low resistance change when stretched. In contrast, material choice for a mechanical sensor should result in a large change in material property when stretched. The two predominant mechanical sensors are either strain sensors or pressure sensors. The performance of stretchable sensors is predominantly characterized by key parameters such as sensitivity or gauge factor (GF), stretchability, signal response and recovery time, hysteresis, durability, and softness. Other desirable attributes can include advanced functional properties such as self-healing capabilities, self-adhesive properties, and optical transparency as well as processing features like ease of fabrication, printability, and scalability.

### 3.1. Sensitivity

The simplest performance metric is signal sensitivity to mechanical deformation, which is often described by the gauge factor (GF). The gauge factor is the slope of the change in signal to the applied strain, as described by

(1) GF=ΔRR0ε or GF=ΔCC0ε

where ΔR or ΔC is the change in resistance or capacitance, R_0_ or C_0_ is the initial resistance or capacitance at ε = 0% strain, and ε is the applied strain. In the case of pressure or mechanical deformation in the normal direction, the pressure sensitivity (PS) would be defined by:

(2) PS=ΔRR0ΔP or PS=ΔCC0ΔP

where ΔP is the change in pressure. Traditionally, metal foils and semiconductors have high GFs over a very small strain range (<5%) with reported ranges of 2–5 for metals and 100–1000 for semiconductors [35]. For nonlinear sensitivity behavior, the GF at the highest strain point is often reported [5,157,158,159]. For piezoelectric materials, the GF is defined by the relative change in electrical current with applied strain where

(3) GF=ΔII0ε

and for pressure sensitivity:

(4) PS=ΔII0ΔP

where ΔI is the change in current and I_0_ is the initial current.

By taking a structural approach, researchers have introduced unique microstructures to enhance sensitivity while retaining stretchable features, resulting in a wide range of GFs. Wan et al. recently demonstrated a wrinkled graphene strain sensor with a GF of 502 and skin-like stretchability across 35% strain (skin strains roughly to 30%) [160]. Pegan et al. achieved a GF of 42 with wrinkled platinum thin films that can strain up to 185% via shrink fabrication [5]. Jeon et al. presented a platinum-based strain sensor with high crack density for measurement of whole-body human motions (>100% strain) [82,161], reporting a gauge factor of 30 at 50% strain at a given thickness of platinum and can extend that stretchability to 150% strain by depositing more platinum [162]. Higher sensitivities are generally achieved by large structural changes to result in increased electrical signal changes. There is an inherent trade-off between sensitivity and stretchability. High stretchability requires the material to maintain structural integrity with elongation, minimizing stress concentrations that could lead to microstructure defects [163]. Most sacrifice some stretchability for higher sensitivity, but a few recent techniques have allowed for a more controlled network of defects in nanomaterials. Amjadi et al. report on a graphite thin film sensor that achieves a sensitivity of 522.6 at 50% strain by exposing the elastomeric substrate to oxygen plasma prior to depositing the thin film, generating parallel microgrooves within the film [161]. By exposing an Ecoflex elastomer to ultraviolet/ozone prior to depositing CNTs, Li et al. are able produce an impressive GF of 1020.2 with large stretchability to 100% [164]. Xin et al. also exhibit high sensitivity and stretchability with laser-engraved CNTs, reporting a GF of 4.2 × 10^4^ at 150% strain [163]. As for intrinsic stretchable conductors, conductive polymers such as PANI elastomer blends have reported GFs of 0.5–1 [165] which is higher than GFs for pure PANI (0.29–0.42) [166].

**Figure 6:**
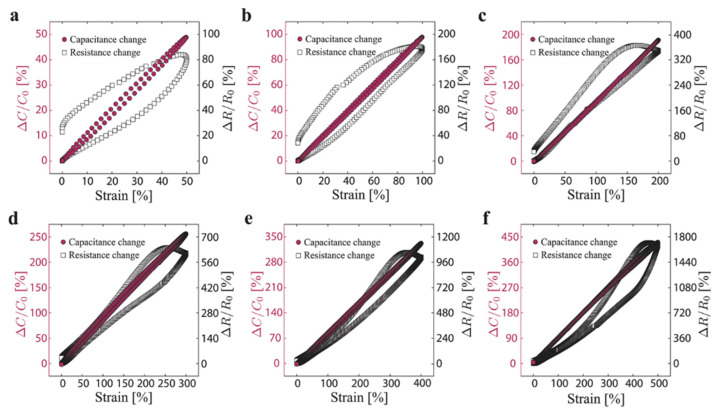
Comparison of capacitive and resistive strain sensor response for carbon black-filled elastomers under different strain amplitudes for (**a**) 50% (**b**) 100% (**c**) 200% (**d**) 300% (**e**) 400% and (**f**) 500% strain. Visible hysteresis can be seen in the resistive response between loading and unloading strain. Reproduced with permission from [167]. Copyright 2017, John Wiley and Sons.

In comparison to piezoresistive sensors, capacitive sensors tend to have relatively low GFs, averaging around GF ~1 but exhibit excellent linearity and little hysteresis with impressive stretchability. Shintake et al. compare carbon black-filled elastomer composite strain sensors for both capacitive-type and resistive-type across an extensive stretchable range (50–500%) [167]. The reported performance comparison is shown in Figure 6. The resulting gauge factors for the capacitive sensors are all closer to 1 for all strain cycles (GF: 0.86–0.98) whereas the resistive sensors exhibit greater variety the strain cycles, increasing with higher strain amplitude. The reported resistive GFs are 1.62–3.37, 2–4 fold greater than that of their capacitive counterparts. While it is rare for capacitive type sensors to have higher sensitivities than 1, recent advances depict composite systems with geometric structures in resistive-based sensors to create electrodes for capacitive systems. For instance, Nur et al. wrinkle ultrathin films of gold electrodes to achieve a gauge factor of 3.05 with stretchability up to 140% strain [168].

Piezoelectric sensors have high sensitivities in comparison to piezoresistive or capacitive sensors but remain highly limited in stretchability. Wu et al. achieve excellent sensitivity with ZnSnO_3_ nanowires/microwires for a piezoelectric material with a GF of 3740 [169]. The strain range, however, is limited to 0.35% strain, making it much more suitable as a flexible device rather than a stretchable one. Dagdeviren et al. have created a lead zirconate titanate pressure sensor with a pressure detection of 0.005 Pa and response time of 0.1 ms [27]. Often, piezoelectric sensors are used as stretchable or flexible energy harvesters [170,171,172,173,174] and applied as pressure sensors [27,175,176] and as actuators in soft robotics [177]. They are particularly promising as energy harvesters to leverage energy from various mechanical deformations like body movement [170,171,172,178,179]. Most piezoelectric materials are rigid, inorganic, and require complicated microfabrication techniques to process into thin films for greater flexibility [180,181]. Common piezoelectric materials for wearable sensors are lead zirconate titanate (PZT), zinc oxide (ZnO) nanowires, and polyvinylidene fluoride (PVDF).

It should be noted that the method of reporting gauge factor may not be entirely representative of the value needed for practical use. For instance, when Amjadi et al. indicate GF as 552.6 at 50% strain, the sensor was also reported as no longer conductive past this point. Moreover, reporting sensitivity values at the point of fracture may also not be a sensitivity that is reproducible for subsequent use. As higher sensitivities are generally achieved by large structural changes to cause increased electrical signal changes, this could also indicate that towards the upper limits of the strain range, the signal is also becoming increasingly unstable due to significant defects and disconnections in the sensing element. Further, a less frequently reported value is sensor resolution which also depends on the intended application in addition to the processor capability. For large scale motion, a sensor with low gauge could be sufficient whereas more subtle motions like facial expression detection may require much higher sensitivity across small strains.

### 3.2. Stretchability

As mentioned often throughout this review, stretchability is a key parameter for the use of soft electronics in wearable systems. Physiologically relevant strain ranges, such as for human motion, may require large deformation of >50% strain [103,162]. For example, bending of the elbow can require upwards of 180% strain whereas strain across the knee can reach 230% while in squat position [181]. It should be noted that these strain values are experimentally determined and can vary from study to study with high sensor placement variation potential on the same joint (e.g., knee bending has been reported as 55% strain [103], 100% strain [182], and 230% strain [181]). This variation also is later seen in the reported values in Table 2 when summarizing sensor performance for motion detection (Section 4.1) which indicates that more detailed reporting beyond basic demonstration is required to help resolve comparable metrics. Future work should include comprehensive investigation into joint motion tracking and detection to determine desired stretchability for the intended application, which may end up being joint specific. 

Moreover, the dynamic range for stretchability across stretchable electronics, can vary significantly and, as previously mentioned, is driven largely by the stretchability of the supporting substrate materials (see Section 2.1). The range for these materials may extend far beyond the ability of the human body. This range may also rely on both the intrinsic stretchability of the conductive material and the interface between the polymer and the conductor. For instance, Park et al. demonstrate a 700% strain range with wrinkled CNT thin films on Ecoflex with two distinct sensing regions from 0–400% strain and 400–700% strain, which approaches the full range of pure Ecoflex (900%) [181]. While these sensors were capable of tracking joint bending without sensor failure, this also brings up the challenge of nonlinearity in stretchable sensor behavior. Researchers have attempted to resolve this by choosing regions of linearity within that range. Future work in this area could involve developing more linearly stretchable polymer materials or turning to capacitive sensors when appropriate.

### 3.3. Hysteresis

Hysteresis is a known phenomenon in elastomeric polymers caused by energy dissipation due to the material internal friction [35]. This can be significant when considering the dynamic loading soft strain sensors undergo in wearable applications. Moreover, large hysteresis leads to irreversible sensing performance with dynamic loading [85,98]. Hysteresis in soft strain sensors are mainly caused by the viscoelastic nature of the polymer but also the interactions with the sensing functional material [82,183]. It can also be dependent on strain load amount and strain rate. Often, hysteresis behavior in soft sensors is observed qualitatively rather than reported quantitatively. Shintake et al. are one of the few to report quantitative values which they call drift error. They define drift error as the error of the sensor reading at 0% strain between before and after the stretch cycle [167]. This parameter, however, does not fully capture the hysteresis behavior that is observed at higher strain points in Figure 6 where there is more pronounced drift between the loading and unloading curves for their resistive sensors. One technique to quantifiably measure hysteresis involves applying a sinusoidal mechanical load and observing the phase lag in the resulting sensor signal which is a similar method utilized with dynamic mechanical analysis of soft polymers. Another method could be to take the area between the loading and unloading curves to give a better picture of the full dynamic domain for a set strain range. In general, hysteresis can be potentially reduced by materials development to minimize the interface mismatch between a polymeric substrate and the active functional material. For instance, Ge et al. introduce an interpenetrating binary-networked hydrogel of polyacrylic acid and polyvinyl alcohol with CNTs with negligible electrical hysteresis due to partial alignment within the hydrogel matrix [184].

### 3.4. Signal Latency Metrics

Electromechanical signal latency metrics such as response time, relaxation time, and signal overshoot of wearable sensors are parameters that are important for practical use as a wearable strain sensor, in particular. Sheridan and Ferrell report that human subject tests consider 45 ms to be the maximum time classified as “no delay” [185]. It is important to note that all polymer-based strain sensors have response delay due to the viscoelastic nature of the polymer; an appropriate response time value for these sensors has been established at a 90% time constant [5,82,186]. Relaxation or recovery time upon releasing mechanical load is also often dominated by the stress relaxation of the polymer and is also prone to a recovery delay. A 90% time constant is also commonly reported for recovery time. Overshoot behavior can also be quantified for polymer-based sensors where a set strain is applied and held constant over time; this signal behavior is often theorized to be dependent on the viscoelasticity of the polymer, GF, and strain rate [183]. Overshoot behavior is also one method of observing the nonlinearity in sensor signal as linearity is often important for stable operation. 

Capacitive sensors tend to exhibit shorter response times than resistive sensors. For example, AgNW capacitive sensors demonstrated response times of 40 ms in comparison to the 200 ms shown in resistive AgNW sensors [82,83]. When strain is released, polymers tend to instantly release stress through mechanical deformations where the internal structure of polymers responds through molecular or molecular segment motions. These internal structure motions may have minimal impact on the dielectric layer whereas small deformations in resistive materials may cause large distances and resulting changes in resistance.

### 3.5. Durability

Stable sensor response to repeated dynamic deformation (often reported from hundreds to tens of thousands of cycles) is representative of the sensor’s durability. The conventional fatigue method is through cyclical uniaxial tensile loading, and ideal behavior would depict stable electrical functionality and mechanical integrity. Again, the elasticity of the supporting substrate is important as it allows the device to bear repeated strain without damage. The number of cycles is most often determined by the potential application and can vary from system to system. Response degradation is attributed to fatigue along with observed plastic deformation of the polymer substrate and eventual fracturing defects within the functional materials at high strains [5,70,82,187]. Related to the discussion on future work to determine appropriate stretchability metrics for practical application (Section 3.2), fatigue studies can help determine if sensor technology is mature enough to withstand prolonged practical application. If fabrication still requires significant labor and effort, and sensors can be easily damaged with mechanical handling, sustaining enough samples for extended human subject testing would be difficult to maintain. Notably, a few thousand cycles are not enough for practical use. While the ideal case would be to extend testing to fatigue failure, for “low cycle fatigue,” an appropriate value to aim for is approximately 100,000 cycles in materials industry according to ASTM International standards (ASTM E606) (American Society for Testing and Materials) [188]. Alternatively, the sensor performance lifetime is also driven by the application and intended use, which can lead to a wide variety in reported cycling values. Improving sensor durability through introducing self-healing capabilities is discussed in the next section.

### 3.6. Self-Healing Capabilities

Ideal wearable sensors should maintain outstanding performance while under significant deformation even in real world conditions including mechanical damage and wear. Recently, there have been considerable interest in development of not only soft stretchable electronics, but also self-healing soft electronics [108,189,190,191,192,193]. Self-healing properties would enhance the service lifetime of these devices and improve their reliability, reusability, and durability, all of which are desirable characteristics in wearable sensors. As conventional elastomers lack self-healing capability, research focus has been on materials development with polymer chemistry driven by biomimicry of the human skin’s natural ability to self-heal from damage. The self-healing mechanism behind these materials have been largely categorized as extrinsic or intrinsic self-healing with extrinsic self-healing mechanism relying on dispersed healing agents to help repair damage. For electrical self-healing, examples of extrinsic self-healing sensors are those that involve liquid metals and ionic liquid-based active components which reflow to allow intermixing of materials at the reconnected interface [194,195]. This extrinsic mechanism is reliable but limited in the number of times it can be healed whereas intrinsic self-healing is dependent upon dynamic reversible covalent or non-covalent bonds which can allow the system to heal repeatedly through reorganization of the polymer matrix and often pertains to mechanical self-healing. Specifically, dynamic covalent bonds can involve Diels-Alder reaction, dynamic hydrazine bonds, disulfide bonds, and metal-ligand coordination whereas non-covalent bonds would include hydrogen bonding, ionic bonding, or supramolecular interactions [11]. Intrinsic self-healing may, however, require external stimuli (e.g., mechanical force or high temperatures) to initiate. Polymer materials that undergo the intrinsic self-healing mechanism tend to be soft and deformable and thus have received much attention for their potential in soft electronics.

**Figure 7:**
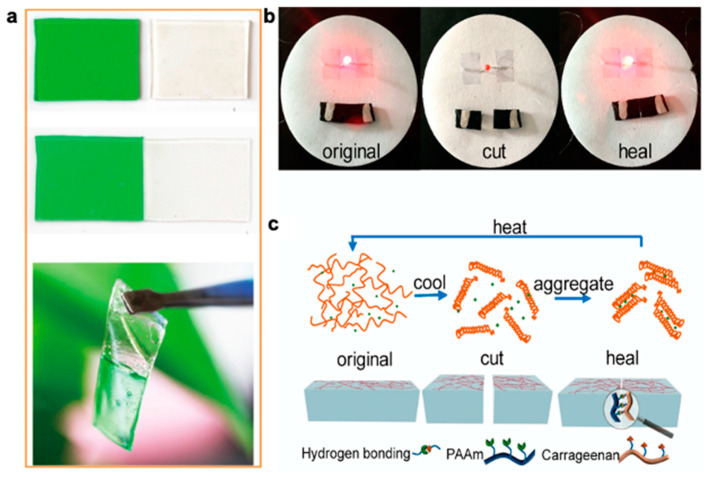
(**a**) Demonstration of a glycerol/hydroxyethycellulose (GHEC) macromolecular elastomeric gel self-healing through presence of dynamic hydrogen bonds [197]. Adapted and reproduced with permission as licensed under the Creative Commons Attribution 4.0 International License. (**b**) Self-healing ability of the dual conductive network hydrogel. (**c**) Schematic of the self-healing mechanism. Adapted with permission from [198]. Copyright 2020, American Chemical Society.

Although these materials can be engineered to have self-healing properties, they tend to have low conductivity. There have been a limited number of self-healing polymer systems applied towards electronics as researchers must take into account both mechanical and electrical properties along with electrical and environmental stability. The design strategy to develop high performance electronics with self-healing capabilities often involves incorporating a conductive filler or conductive polymer into the self-healing polymer matrix which would require high compatibility between both materials for simultaneous electrical and mechanical self-healing [196]. A representative demonstration of a couple self-healing polymers healing via different mechanisms is shown in Figure 7.

Further, for stretchable electronics, the challenge lies in maintaining high electrical conductivity, self-healing capabilities, and stretchability as self-healing conductive materials remain largely limited in stretchability (<100%) [199,200]. One approach to increase stretchability involves constructing hybrid materials composed of conductive fillers, conductive polymers, and intrinsic self-healable elastomers. Li et al. utilize AgNWs, modified PEDOT, and a Diels-Alder elastomeric copolymer to bridge electrical conductivity, self-healing, and stretchability to 100% strain [201]. Han et al. modify a commercially available epoxidized natural rubber with polydopamine (PDA) and crosslinks reversible catechol-Fe^3+^ coordination bonds and take a hierarchical structure design approach with CNTs to fabricate a sensor with high sensitivity, pristine (GF 37.7) and self-healed (GF 16.2), and low detection limit (0.05% strain) [191]. 

Another approach is to develop new conductive polymer complexes entirely. For example, Oh et al. report a metal-ligand coordination self-healable device that relies on a semiconducting polymer, poly(3,6-di(thiophen-2-yl)diketopyrrolo[3,4-c]pyrrole-1,4-dione-alt-1,2-dithienylethene) with 10 mol% 2,6-pyridinedicarboxamine moieties (DPP-TVT-PDCA), for its good charge carrier mobility combined with poly(dimethylsiloxane-alt-2,6-pyridinedicarbozamine) (PDMS-PDCA) that can mechanically strain to 1300% and self-heal within 24 h. The reported gauge factor was 5.75 × 10^5^ at 100% strain, which is among the highest reported for semiconducting strain gauges [202]. Resistivity changes in semiconductors are due to reversible microstructure changes in the material which result in far higher sensitivities that can enable very small strain detection [203]. That being said, while the sensitivity strain curve was not provided for this material, the stress-strain curve indicates plastic deformation beyond 100% despite ductile behavior that allows it to continue to mechanically strain to 1300%. The effective elastic region appears to be 0–100% strain, potentially making both reported values less meaningful for strain sensing in practical application. Wang et al. developed a ternary polymer composite of PANI, polyacrylic acid (PAA) and phytic acid (PA) that relies on hydrogen bonding and electrostatic interactions for self-healing and is capable of straining to 500% with electrical conductivity of 0.1 S cm^−1^ and >99% healing efficiency in 24 h [145]. In comparison, Lu et al. synthesize PANI and PA with poly(2-acrylamido-2-methyl-1-propanesulfonic acid) (PAAMPSA) to enhance stretchability to 1935% strain and GFs ranging from 0.62–1.31 capable of self-healing without external stimuli [190]. Li et al. craft an “all-in-one” molecular network design by introducing dynamic hydrogen bonds into polymerizable deep eutectic solvent-based elastomers with either acrylic acid/choline chloride (AAm/ChCl) or maleic acid/choline chloride (MA/ChCl) molecules. This results in self-healing, transparent, and ionically conductive (conductivity 4 × 10^−4^ S cm^−1^) elastomers that can self-heal within 2 s without other external stimuli and strain to 450%. Impressively, these conductors remain stretchable from subzero to high temperature and enable human monitoring over a wide range of temperatures (−23 to 60 °C) [204].

Other researchers turn to formulation of self-healing stretchable hydrogels, and, as with conventional hydrogels (see Section 2.1.2), filler, conductive polymers, and ionic elements can also be incorporated into the polymer matrix of self-healable hydrogels. Cai et al. introduce a dynamic crosslinked hydrogel of polyvinyl alcohol (PVA) and Borax that can then be homogenously mixed with CNTs, graphene, or AgNWs, strain to 1000% and self-heal within 3.2 s. They report GF of 1.51 for CNTs/hydrogel [12]. Zhu et al. facilitate PANI-containing conductive hydrogel networks through preorganized α-cyclodextrin-containing Poly(N-isopropylacrylamide) (PNIPAM) with homogenous, interconnected macropores, allowing for ideal integration between PANI and PNIPAM. This conductive self-healing hydrogel exhibits high conductivity (0.64 S cm^−1^) and ultimate tensile strain of 490% [205]. Lei et al. introduce a supramolecular mineral hydrogel composed of amorphous calcium carbonate (ACC) nanoparticles physically crosslinked by PAA/alginate chains that is sensitive to small pressure changes up to 1 kPa and tensile strain range of 100% and is capable of autonomous self-healing within 20 min at room temperature [28]. Ge et al. introduce another “all-in-one” self-healing and anti-freezing binary-networked hydrogel of PAA and PVA capable of stretching to 550% strain that relies on metal-coordinated bonds and tetrahedral borate interactions for self-healing and maintains stretchability even under −25 °C. This hydrogel is turned into a strain sensor through dispersion of CNTs into the hydrogel matrix with a GF ranging from 0.66–1.61 within a 100% strain range. This sensor also displays negligible electrical hysteresis and has a response time of ~31 ms [184].

Note that all the previously reported self-healing electronics above are based on composite systems where the percolating network of the conductive elements can easily recontact in order to recover conductivity. Further, the conductivity of such materials remains lower than that of conventional conductors so while the other outstanding properties are noteworthy, the conductivity of electrode materials need to be >1 S cm^−1^ for practical applications [196].

### 3.7. Self-Adhesive Abilities

Wearable sensors require attachment to the body, often via the addition of medical tapes. While a lot of focus has been devoted towards sensor development, less has been on adhesives for soft stretchable sensors as most will reach for readily available biocompatible athletic or medical tapes. Incompatibility between the adhesive and sensor may contribute to mounting complications and premature delamination, causing signal instabilities and inaccuracies. It should be noted that the Rogers group has also made meaningful progress in biocompatible adhesives for their electronic systems and have found silicone-based adhesives more gentle and safer for neonatal skin which is more fragile than adult skin [206]. Longer wear times would require higher adhesion to prevent lifting along the edges with wear [207]. Ideally, stretchable electronics would maintain good conformal contact to the skin—which is curvilinear, coarse, and dynamic, without interfering with natural movement during the use lifetime. Mounting would also be, preferably, simple and unobtrusive. Stretchable sensors with self-adhesive abilities could simplify the process and help promote more stable signal detection by ensuring conformal contact. One simple method to modify the adhesive properties of PDMS, a widely used silicone-based elastomer, is to add small amount of an amine-based polymer, ethoxylated polyethylenimine (PEIE), into the base and crosslinker mixture. As a low viscosity material, it can be easily integrated into the mixing process. Varying the PEIE concentration allows researchers to tune the mechanical characteristics as the PEIE additive will soften mechanical modulus and increase stretchability and adhesion force of the adapted PDMS elastomer [208]. Other chemical modifications involve polymerizing supramolecular elastomers with conductive polymers that display both self-healing and self-adhesive properties [209].

**Figure 8:**
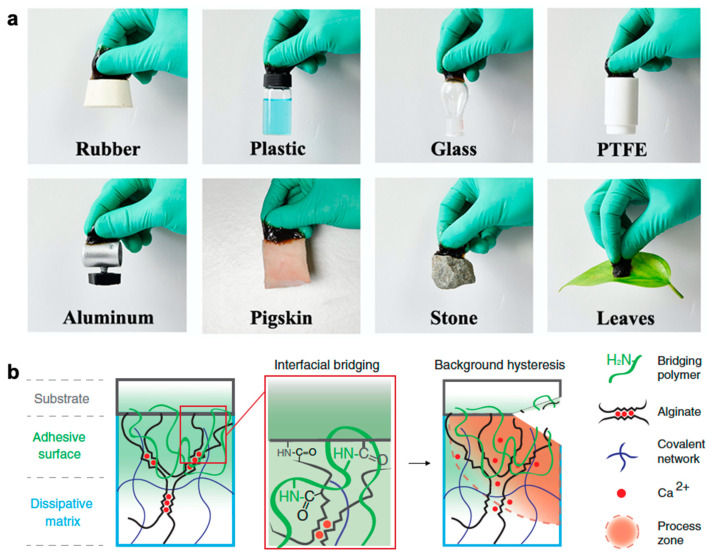
(**a**) Demonstration of PDA-based hydrogel adhesion to various materials. Adapted with permission from [210]. Copyright 2019, American Chemical Society. (**b**) Design schematic for a hydrogel-based tough adhesive inspired by slug mucus which shows how the bridging polymer can be absorbed to the tissue surface through electrostatic attractions and allow for covalent bonding [214]. Copyright 2017, The American Association for the Advancement of Science.

Others have turned to modifying hydrogels to be adhesive, and, similar to instilling self-healing properties, introducing adhesive properties into hydrogel formulation can also come at the expense of mechanical toughness. Ideal hydrogel sensors would have self-healing capabilities, stretchability, adhesive properties, and sufficient conductivity for practical use. As such, certain self-healing mechanisms may also contribute to self-adhesion capabilities as well. In particular, PDA is a synthetic polymer inspired by mussels which exhibits strong interfacial adhesion strength, and, when incorporated into the hydrogel matrix, imparts self-adhesive abilities to self-healable hydrogel-based sensors [210,211,212,213]. An example of PDA-based hydrogels exhibiting adhesion to various materials is shown in Figure 8a. Another biomimetic tough adhesive for biological application is inspired by the defensive mucus secreted by slugs. Li et al. fabricate two-layered tough adhesives that contain: (i) an interpenetrating positively charged polymer and (ii) a dissipative hydrogel matrix to allow for adhesion to wet negatively charged surfaces of tissues and cells and formation of covalent bonds across the interface [214]. A schematic of their adhesion mechanism with desired design criteria can be found in Figure 8b. Specifically, they include a bridging polymer with positively charged primary amine groups (chitosan, polyallylamine) similar to the amine groups found in slug adhesive which are believed to play a large role in its adhesion. They found alginate-polyacrylamide to have high mechanical toughness and to most effectively dissipate energy to prevent background hysteresis. These tough adhesives demonstrate strong adhesion to porcine skin, cartilage, heart, artery, and liver.

## 4. Applications

Soft stretchable sensors have been demonstrated for motion detection with great potential for rehabilitation, continuous healthcare monitoring, and use in wearable consumer interface such as virtual reality and interactive gaming. Low strain detection can be used in conformal pressure sensing and tactile motion for soft robotics [7,215] whereas high strain detection allows for body-interfaced motion detection and athletic performance monitoring. Also, soft sensors that retain high conductivity with high stretchability but demonstrate low signal sensitivity can be leveraged as soft interconnects for wearable electronics [216,217]. Here, we summarize three main applications for stretchable electronics with motion detection being the most commonly demonstrated, healthcare monitoring having the greatest need, and consumer use with advances in human-machine interfaces as a highly desired avenue. It is also important to note that all the soft sensors discussed here are noninvasive and are not implanted within the body.

### 4.1. Motion Detection and Rehabilitation

**Figure 9:**
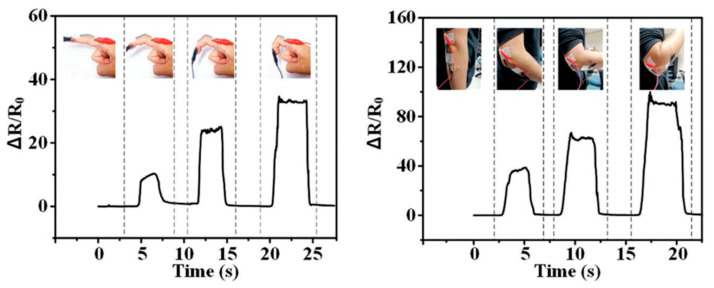
Demonstration of a resistive strain sensor using overlapped CNTs to track bending of the finger and elbow, respectively. Reproduced with permission from [221]. Copyright 2019, John Wiley and Sons.

**Figure 10:**
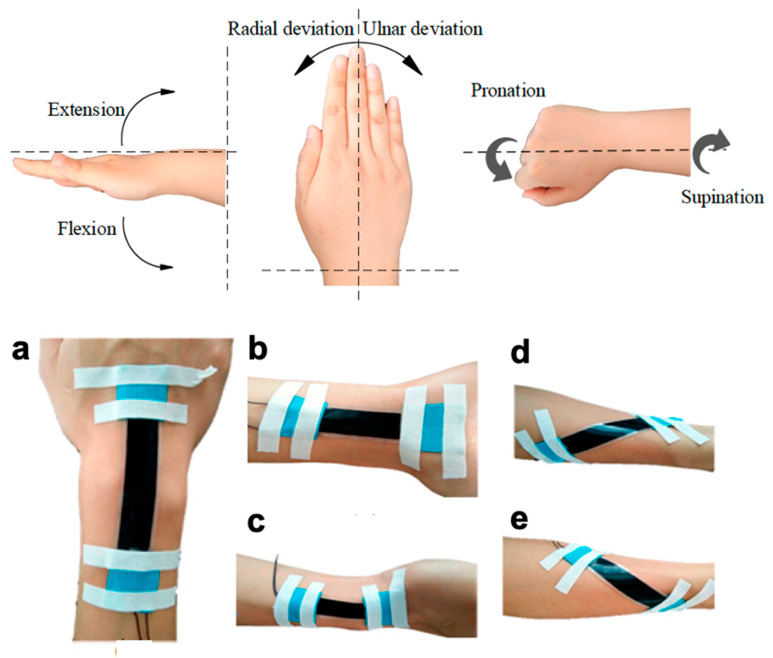
Decomposition of possible wrist motions for the wrist joint along with corresponding placement of a capacitive motion sensor for tracking of (**a**) flexion (**b**) extension (**c**) Ulnar deviation (**d**) pronation (**e**) supination [222]. Reproduced with permission as licensed under the Creative Commons Attribution 4.0 International License.

Abnormal body movement can be symptomatic of underlying diseases that affect the nervous system. Wearable sensors to monitor range of motion can have impact on early detection. Moreover, having accurate motion detection sensors can help assess the effectiveness of rehabilitation exercises and help guide future rehabilitation treatment [218]. These types of sensors could also be applied towards athletic sports performance with detection of various exercises and motion patterns along with gait and balance analysis and joint specific motions. As human motion can range from more subtle movements such as swallowing [219], respiration [220], vocal phonation [7], and facial expressions [3,4] to large scale motions like joint movement of the knee, elbow, hand, or fingers [181,182,221] (as demonstrated in Figure 9), wearable strain sensors developed for motion detection must have high sensitivity as well as a large dynamic range. As previously stated, these sensors must also be able to maintain conformal contact with the curvilinear planes of the body to allow for accurate monitoring. Researchers have developed high performing sensors tailored for specific motions or areas of interest at the start. An example of stretchable strain sensing for tracking the complex motion of the wrist joint with a soft capacitive sensor through sensor placement is shown in Figure 10 [222] while Table 2 summarizes representative strain sensors for motion detection from the perspective of materials, conductive type, sensing type, sensitivity (GF), sensing range, and application.

**Figure 11:**
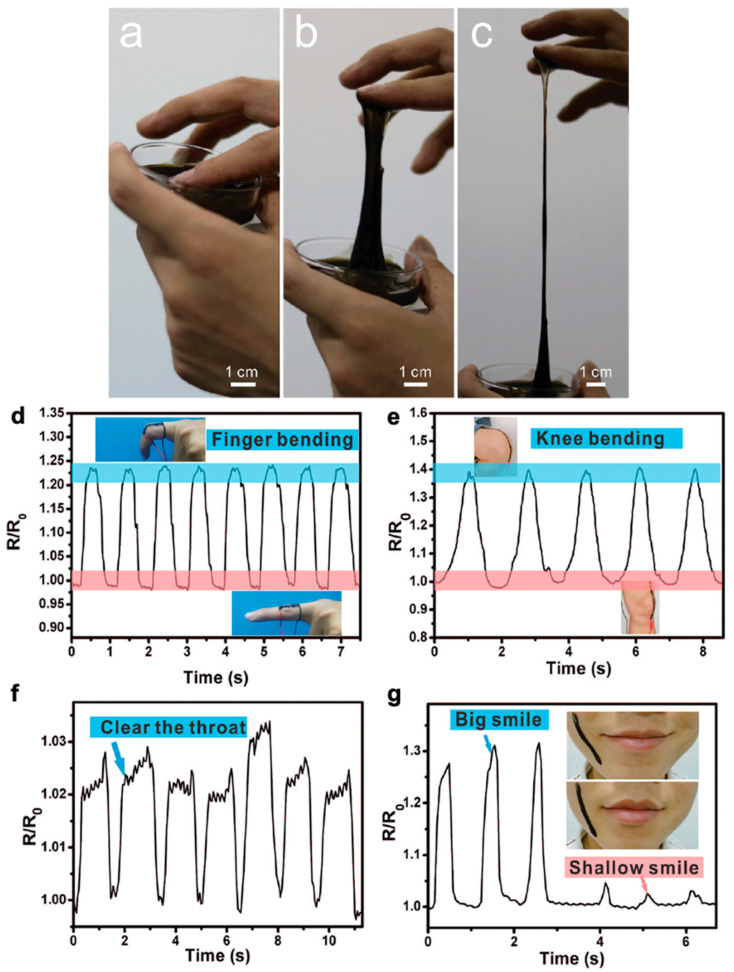
(**a**–**c**) Demonstration of PVA/PDA hydrogel compliance, fluidity, and self-adhesion to fingertip. Motion detection demonstration of the PVA/PDA hydrogel with (**d**) finger bending, (**e**) knee bending, (**f**) throat movement, and (**g**) smiling. Reprinted with permission from [230]. Copyright 2018 The Royal Society of Chemistry.

To highlight the wide range of this application category, some recent work on newer classes of materials have been reviewed here. For the detection of micro-deformations such as facial expressions and swallowing, ionic hydrogels can be an attractive material choice for strain sensors due to their high stretchability, compliance, and self-adhesion capabilities. This self-adhesive property is advantageous as it increases the magnitude of effective contact area between the sensor and skin surface for more accurate detection and removes the need for additional adhesive tape or binding to secure the sensor to the body. A PVA/PDA hydrogel blend reported by Liu et al. is capable of detecting ultralow strain of 0.1% without the need for high sensitivity and even demonstrates excellent signal resolution of 0.1% strain below 0.5% strain range [230]. Conductivity is attributed to the abundance of Na^+^ in the water of the hydrogel, leaving this sensor free of a conductive network created by conductive fillers. This lack of friction between conductive elements and polymer matrix allows the conductive network to recover during stretch-release cycles and would also minimize electromechanical hysteresis (not reported). The sensing performance is reliant on the shape changes of the PVA/PDA sensor and allows for great sensing linearity (R^2^ = 0.99). While this hydrogel sensor also has full dynamic range to 500%, which would encompass potential large motion such as knee bending demonstrated in Figure 11e, the sensor’s high linearity is a more significant factor in its detection capability. This also suggests that high gauge factor is not necessarily the only significant sensor metric to determine performance for stretchable electronics.

**Figure 12:**
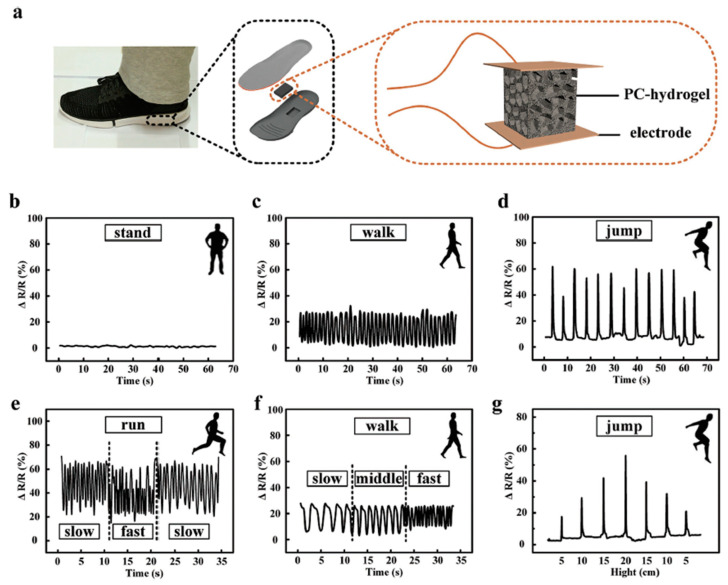
(**a**) Schematic of strain sensors based on PC-hydrogel and sensor placement in running shoes with detection under various actions: (**b**) standing (**c**) walking (**d**) jumping. (**e**–**g**) Varied resistance response of the PC-hydrogel strain sensor to different speeds for running and walking and jumping to different heights. Reproduced with permission from [149]. Copyright 2018 John Wiley and Sons.

Another approach towards fabricating conductive hydrogels involves creating a composite that consists of a conductive polymer and hydrogel by diffusing the conductive polymer monomer into a supporting hydrogel matrix as discussed in Section 2.1.2. Gu et al. fabricate a macroporous conductive hydrogel (PC-hydrogel) by incorporating a stiffer conductive polymer (Ppy) into the macroporous structure of a soft hydrogel (poly(ethylene glycol)-dimethacrylate (PEG-DMA)) [149]. By leveraging the properties of the hybrid networks, these PC-hydrogels have high fatigue resistance and electrical conductivity to allow detection of compressive strain from 10–50%. This work emphasizes the fatigue resistance of their sensors and leaves other sensor performance metrics largely unreported. It is likely the interfacial interaction between the conductive polymer and the porous hydrogel matrix, along with the porous nature, that enhances the deformability of the sensor. Thereby, it is possible to detect a variety of physical actions such as standing, walking, running, and jumping shown in Figure 12 and demonstrate potential for gait analysis and sports performance applications [149]. While this work highlights another mechanism to track motion, the demonstration mainly serves as an initial proof-of-concept.

**Figure 13:**
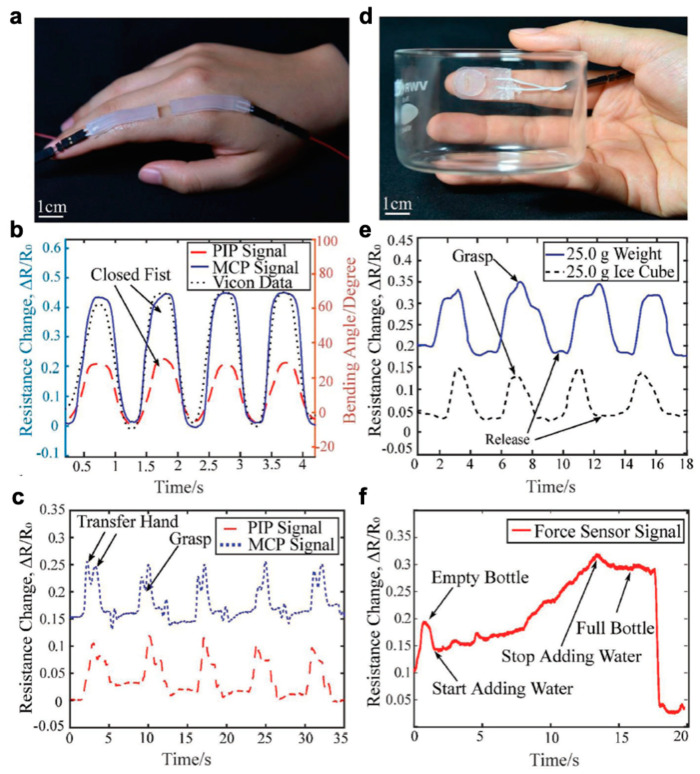
Demonstration of various functionalities as wearable sensors for hand motion detection with strain sensing along the proximal interphalangeal (PIP) joint and metacarpophalangeal (MCP) joint (**a**–**c**) and force sensing at the index fingertip for picking up items of different temperature (**d**,**e**) and force sensing of dynamic motions (**f**). Reproduced with permission from [234]. Copyright 2018 John Wiley and Sons.

Ionic soft sensors are not only limited to ionic hydrogel construction and can be similarly fabricated as liquid metal-based strain sensors which are often patterned as channels within a silicone substrate. For instance, silicone-based sensors composed of biocompatible conductive liquid, potassium iodide and glycerol (KI-Gly), are introduced by Xu et al. for strain and force detection. These sensors exhibit low hysteresis along with high linearity and report GF of 2.2 [234]. These performance metrics suggest high electromechanical coupling between the ionic fluid and the hydrogel substrate. They demonstrate hand motion detection and force sensing associated with different actions (Figure 13). This would have potential application in motion capture and future human-machine interaction, but, again, requires further studies for practical application.

**Figure 14:**
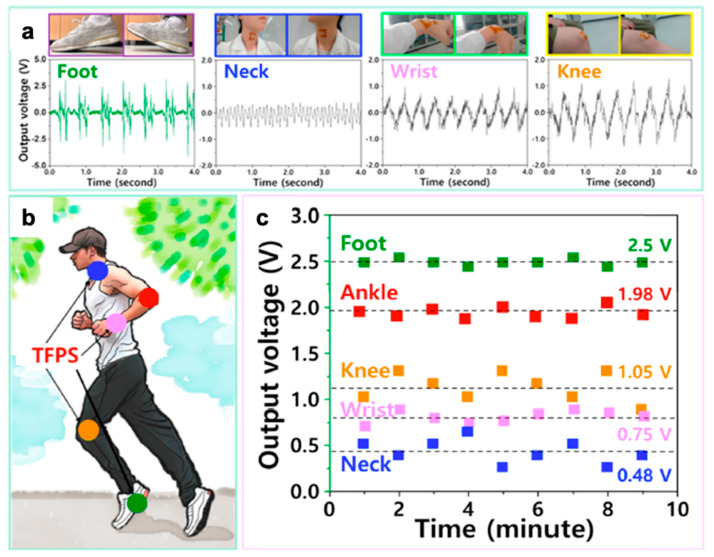
(**a**) Output voltage demonstration of TFPS with movement of foot, neck (voice vibration), wrist, and knee. (**b**,**c**) Schematic of device placement on a running individual along with stability tests at the corresponding locations. The statistical results were recorded nine times for 10 min under identical conditions. Reproduced with permission from [235]. Copyright Elsevier 2018.

Another approach to motion detection is to utilize soft piezoelectric sensors as a means to capture the physical motion energy of the body. As piezoelectric sensors will generate an electrical signal when undergoing mechanical motion, these devices also have potential as energy harvesters for wearable sustainable electrical power generators driven by different types of human movement. Kim et al. demonstrate a transparent and flexible piezoelectric sensor (TFPS) system composed of biocompatible boron nitride nanosheet (BNNS) dispersed in PDMS to not only generate energy, but also measure human movement as shown in Figure 14 [235]. Dahiya et al. fabricate another nanocomposite-based stretchable nanogenerator (SNG)—by encapsulating zinc oxide (ZnO) nanowires in a parylene C polymer matrix on a PDMS substrate—which has the ability to detect the bending of the index finger [170].

Overall, the wide range displayed in Table 2 indicates that while researchers have had great success in fabricating a diverse plethora of stretchable sensors for motion detection as a broad category, more work must be done to further refine meaningful practical use. As previously mentioned in discussion on stretchability (Section 3.2), motion specific strain values are determined experimentally and can differ among the various studies. Moreover, motion detection capabilities are often left merely as basic demonstration for potential application. Further comprehensive investigation into the practical application is necessary to better understand movement measurement, motion differentiation, calibration needs, and the impact of placement variation. Section 3.1 covered sensor sensitivity and summarized the large research focus devoted towards increasing gauge factor for improved performance. The research discussed in this section (Section 4.1), however, suggests that although high sensitivity can be significant, it is not the only significant factor and that future progress should involve other notable factors for sensor performance such as signal resolution, linearity, and lack of hysteresis.

### 4.2. Biomedical and Healthcare Monitoring

Soft stretchable sensors also present a promising avenue for remote and personalized healthcare monitoring outside of a centralized medical facility where health information is limited to a singular moment within a visit. Physicians often look at vital signs such as body temperature and heart activity (i.e., electrocardiogram (ECG) for heart rate) as indicators of health as they closely relate to physical and mental health. Particularly for patients with known health issues, real-time continuous monitoring would provide further insight on physiological health with day-to-day activity in a natural setting, allow for the establishment of a health baseline, and can potentially alert the patient and physician of abnormalities that would require further medical attention. Wearable soft sensors have been shown capable of detecting vital signals such as body temperature [236,237], heart rate [238,239], blood pressure [7,239], and respiration [5,6] and can offer detection in a more natural manner along with real-time monitoring capability.

**Figure 15:**
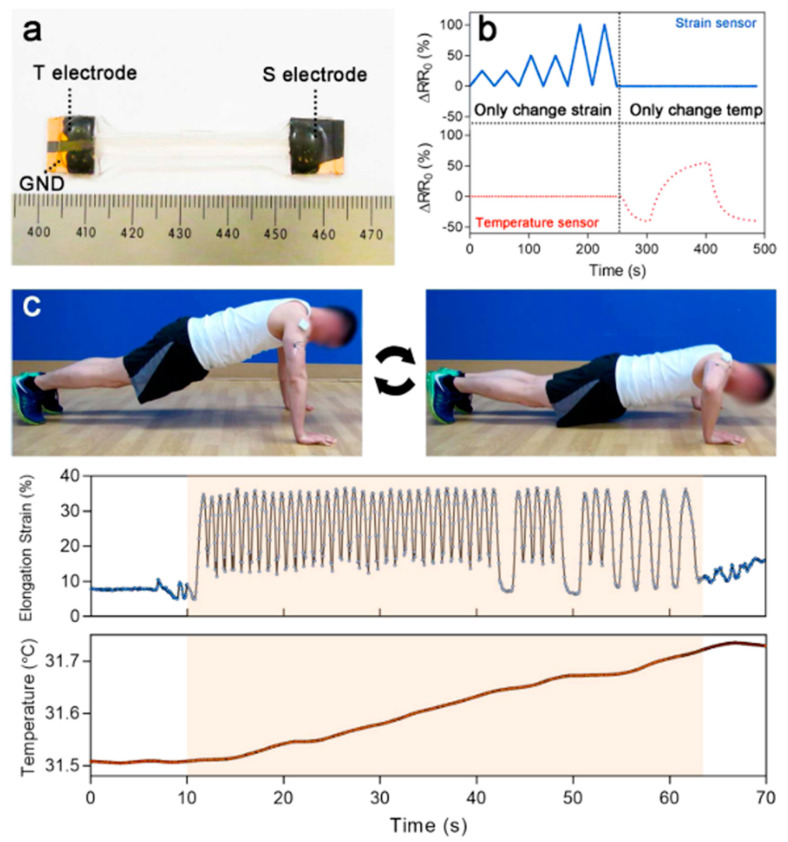
(**a**) Image of an iono-elastomer thermomechanical dual-responsive sensors with T (temperature) and S (strain) outputs and GND acting as a ground for the two electrodes. (**b**) Decoupled signals in response to first mechanical stress and then coming into contact with a cold and hot object. (**c**) Human subject undergoing high-intensity anaerobic exercise with real-time strain and temperature responses captured by the sensor depicted in (**a**). Reprinted with permission from [242]. Copyright American Chemical Society 2018.

**Figure 16:**
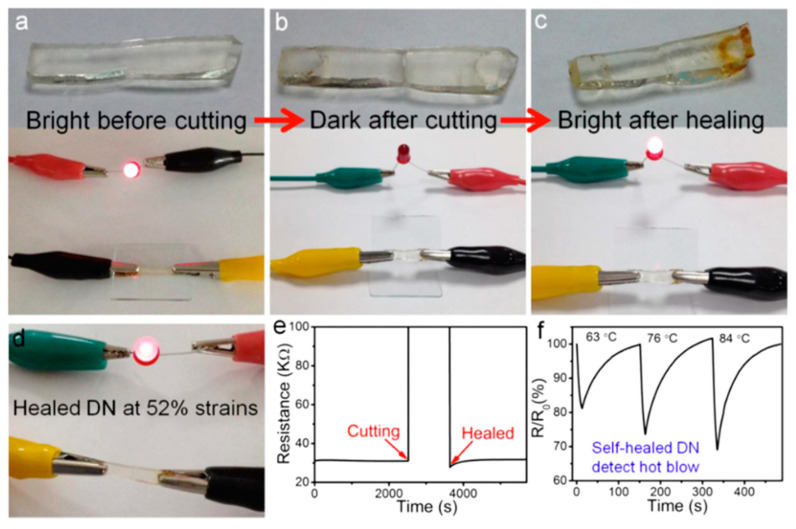
(**a**–**c**) Demonstration of the self-healing conductivity for a double network hydrogel before and after cutting and after self-healing. (**d**) The double network hydrogel remains conductive at 52% strain after self-healing. (**e**) Resistive time-evolution of the self-healing process. (**f**) Demonstration of dynamic response to different temperatures after self-healing. Reprinted with permission from [22]. Copyright 2018 American Chemical Society.

Temperature is one of the first vital signs measured as body temperature can be indicative of infection or low blood flow in cases of elevated or low core temperature, respectively. Being able to monitor temperature would allow for better management of medical conditions and early detection of infections. Generally, temperature sensors rely on a thermoresistive sensing mechanism where the resistance changes with temperature and is largely dependent on the material’s intrinsic temperature coefficient. They are often placed on the arm or chest where the measured temperature from the skin surface is typically lower than core body temperature due to surface exposure to ambient conditions. The range of temperature on the skin is typically 31.1 to 36.5 °C [240]. Stretchable temperature sensors can rely on the thermoresistive sensitivity of conductive thin films [237]. Alternatively, nanocomposites can be used to enhance the temperature sensitivity where structural changes from the interface between conductive fillers have a contributing factor [236]. Researchers have also explored temperature sensing in ionic conductors. For instance, Wu et al. developed a thermistor composed of double network ionic hydrogel (polyacrylamide (PAM)/carrageenan) that is highly sensitive (upward of 2.6%/°C at 200% strain) [22]. This high sensitivity is attributed to the ionic transporting behavior as ionic mobility increases with temperature [241]. This sensor has a full dynamic range of 330%, and the researchers theorize that increased strain aligns the ionic conductive pathways to allow for higher conductivity under a stretched state to increase thermal response. The reported minimal detectable temperature change of this thermistor is 0.77 °C [22]. Moreover, being able to differentiate between a signal caused by temperature or by strain would be crucial in a dynamic environment. To decouple the two signals, a proposed solution is to calibrate a stretchable thermistor by adding a temperature-insensitive strain sensor for purely strain detection or vice versa where a strain sensor is paired with a strain-insensitive temperature sensor. Xie et al. apply this technique to their temperature iono-elastomer, namely crosslinked self-assembled triblock copolymer micelles in ionic liquids, in a demonstration of the response tracking during high-intensity anaerobic exercise in Figure 15 [242] where the iono-elastomer temperature sensing portion was immobilized from strain. Other stretchable ionic temperature sensors also exhibit high linearity, high transparency, self-healing ability (as demonstrated in Figure 16) and can maintain stable conductivity under large deformations [133,243].

**Figure 17:**
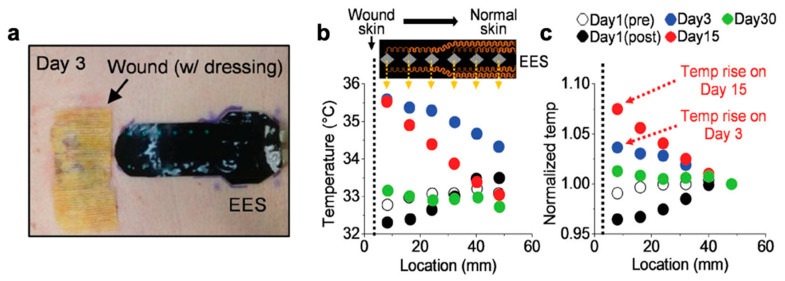
(**a**) A representative image of the EES mounted lateral to post-surgical suture wound on Day 3 with corresponding temperature changes measured over a month of healing (**b**,**c**). Reproduced with permission from [21]. Copyright John Wiley and Sons, 2014.

Temperature sensors have also been applied locally to monitor wound healing where prolonged temperature increase of at least 1.11 °C could be a sign of infection and metabolic activity changes [244]. For example, Hattori et al. created a skin-like epidermal electronic skin (EES) system that can be laminated to the wound site and record real-time temperature and thermal conductivity of the skin [21]. The EES leverages a fractal patterned copper mesh interconnecting an array of six sensors/actuators that are first laminated onto a silicone membrane before being encapsulated with another silicone layer. The fractal pattern allows the copper to strain to 30% which is comparable to the amount of strain tolerated by skin [245]. This device performance was calibrated with an IR camera before being used to track the wound healing of a granulated wound and post-surgical suture recovery where it accurately captured the extended inflammation phase with elevated temperature coupled with a stable thermal conductivity during a prolonged period (as shown in Figure 17). In this scenario, silicones and silicone-adhesives would actually be preferable as those are more appropriate for delicate skin.

Blood pressure is another vital sign that is indicative of both heart activity and overall health where the systolic (maxima) and diastolic (minima) values from a simple inflatable arm cuff (sphygmomanometer) are used to assess health and potential underlying diseases. The blood pressure of a healthy individual has been established at below 120/80 (systolic/diastolic values) [221] whereas values above that are categorized as hypertension, which is one of the key risk factors for cardiovascular disease, stroke and kidney failure and premature mortality and disability [246]. Moreover, these blood pressure measurements are dependent on stationary equipment and cannot offer long-term continuous monitoring. This can lead to asymptomatic cardiac conditions remaining undetected—particularly as most hypertensive patients remain unaware of their condition—until an acute health state such as a heart attack occurs. In addition to lack of long-term monitoring capabilities, this method offers no insight into the pressure pulse waveform which can be used to prognose those cardiac conditions when blood pressure variability has been reported as a relevant prognostic factor [247].

**Figure 18:**
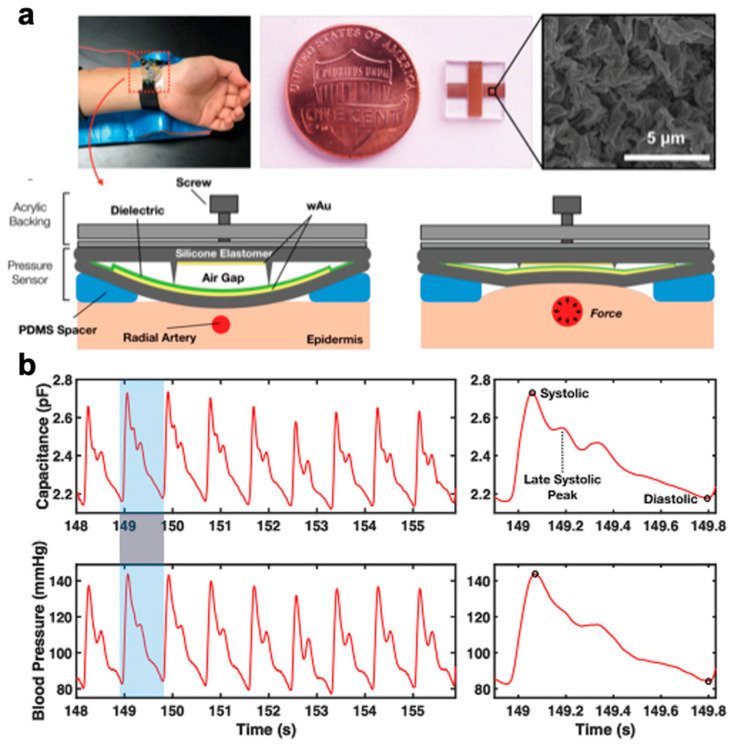
(**a**) Demonstration of wrinkled gold capacitive blood pressure sensor placement and set-up along with (**b**) corresponding arterial pulse waveforms for beat-to-beat measurements. Reproduced with permission from [8]. Copyright 2019 John Wiley and Sons.

Having compliant wearable sensors can help bridge that gap with the development of strain sensors with high pressure sensitivity and low limit of detection to readily capture the pulsatile waveform in a noninvasive manner. These novel soft pressure strain sensors tend to be either capacitive or piezoelectric based—where compression of a soft dielectric layer would cause a change in capacitance or induce an electrical voltage across the device—as these transduction mechanisms have rapid response times and are geometrically suited for compression-based detection. For piezoelectric pressure sensors, Dagdeviren et al. have shown that ultrathin layers of PZT can withstand 30% strain [27,248,249] which is an important consideration as this strain level is on par with the stretchability of the skin and bulk PZT only allowed for ~1% strain. Piezoelectric sensors have excellent signal sensitivity but will require operation under dynamic sensing modes and may depend on complicated microfabrication for signal accuracy. Kim et al. report a quick response time of 10 ms with pressure sensitivity of 0.148 kPa^−1^ and pressure range up to 10 kPa for a wrinkled gold capacitive sensor for beat-to-beat blood pressure detection (Figure 18) [8]. This sensor construction enhances the pressure sensitivity which is a critical parameter to measure arterial pulse pressures, and its quick response time allows for high fidelity detection of the radial arterial pulse waveform.

**Figure 19:**
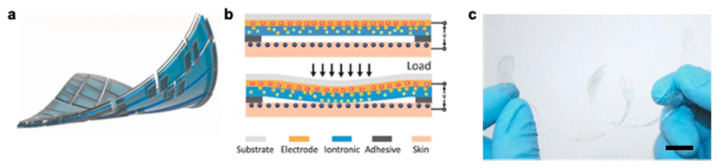
(**a**) Perspective view and (**b**) cross-sectional view of the epidermal-iontronic interface device. Reproduced with permission from [122]. Copyright 2018, John Wiley and Sons. (**c**) Photo of an iontronic pressure sensor array. Scale bar is 1 cm. Reproduced with permission from [125]. Copyright 2015, Springer Nature.

Further, high device sensitivity, high noise immunity, and conformal packing and attachment to the human body are highly sought-after features for blood pressure detection. As mentioned in Section 2.3.2, ionic conductors can form an electric double layer when paired with electronic conductors. Pressure-induced capacitive change can be significant at this interface and can substantially overcome long-standing parasitic noise issues [250]. Advancements in ionic materials and iontronic sensing mechanisms have allowed researchers to explore this phenomenon, but electronic designs and polymer materials challenges have slowed conversion into wearable form. Pan’s group has made considerable strides in this area, presenting skin-interfaced iontronic pressure sensors as shown in Figure 19. Xu et al. also leverage a capacitive electronic double layer with a combination of ionic hydrogels and metal nanofibers for physiological sensing [223].

Respiration is another primary vital sign routinely monitored as insufficient oxygen intake can have serious and even fatal risk for a patient. Abnormal breathing patterns can be indicative of underlying conditions such as sleep apneas, asthma, chronic pulmonary disease (COPD) which can severely impact a person’s quality of life. Common clinical methods for determining respiratory health range from pulmonary function tests conducted with spirometry to monitor airflow to plethysmography to assess lung volume. However, these assessments offer a singular evaluation within a clinical setting and may not be representative of a patient’s respiratory state under normal activity in an outside environment. Moreover, methods like spirometry require a patient to breathe maximally into a mouthpiece which can be an uncomfortable and challenging maneuver, especially for those with a potential pulmonary condition. These maneuvers are difficult to ensure accurate readings and do not allow for long term assessment of a patient’s respiratory health. Plethysmography track the physical expansion and contraction of the chest and abdomen during breathing but rely on inductive belts that are cumbersome and prone to slipping.

**Figure 20:**
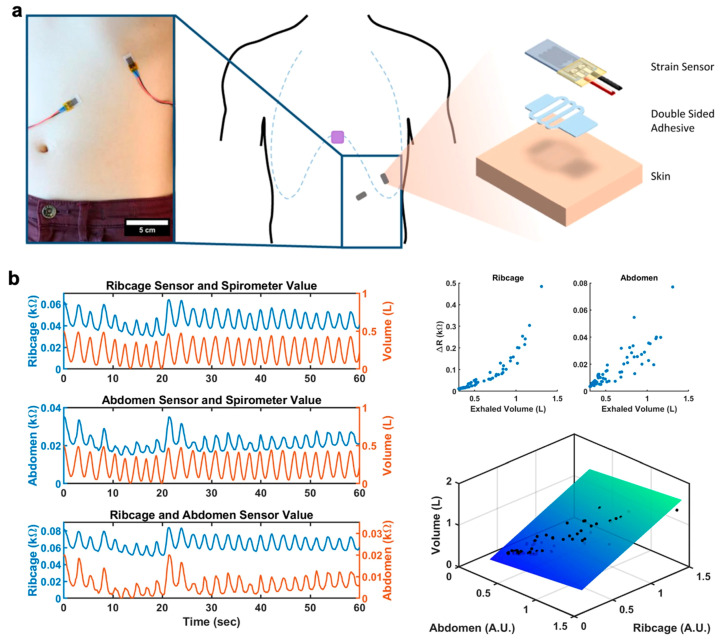
(**a**) Demonstration of sensor placement for ribcage and abdomen along with attachment setup. (**b**) Representative signals from ribcage and abdomen plotted with simultaneous respiration volume along with scatterplots for each [6]. Adapted and reproduced with permission as licensed under the Creative Commons Attribution 4.0 International License.

Stretchable strain sensors can offer detection of respiration through easy placement on the body and physically expand and contract along with the chest wall movement. Advancements in stretchable conductors have allowed for more comfortable, unobtrusive strain sensors that can maintain conformal contact and readily be mounted onto the body with minimal discomfort. These soft wearable strain sensors have been developed with novel nanomaterials and designs into a bandage-like form factor with appropriate signal sensitivities for subtle motions and low strain detection capabilities. Atalay et al. demonstrate a capacitive strain sensor with laser-treated microstructure metal electrodes and silicone elastomer as a dielectric and a strain sensitivity of 0.90 for a linear range of 85% strain capable of detecting respiration rate on the abdomen with a detection resolution of extension below a millimeter [225]. This sensor type is stretchable to 250% strain but displays a nonlinear signal past 85% strain. Pegan et al. also demonstrate respiration rate detection capabilities with a piezoresistive strain sensor with a wrinkled metallic thin film where the hierarchical wrinkle features allow for greater dynamic strain range while maintaining signal sensitivity for the necessary detection strain range (GFs ranging from 0.85 to 2.64 for up to 40% strain) [5]. Chu et al. use these previously reported wrinkled metal thin film piezoresistive sensors placed on the chest and abdomen to validate respiration rate and respiration volume against a clinical continuous spirometer (as shown in Figure 20) [6]. While most small sized wearable sensors report on respiration rate, this is the first reported for determining respiration volume with high fidelity. In addition to nano-and micro-structured thin films, others propose nanomaterial composites to achieve the necessary strain sensitivity for respiration detection. Ho et al. report an ultra-sensitive strain gauge based on high aspect ratio nanowires using a hybrid percolating network of both “soft” AuNWs and “rigid” AgNWs interspersed into PDMS to tune to strain detection from 0.05% to 70% with an extremely high GF of 236.6 in the low strain regime (<5%) [251]. A major point, however, for this paper was to maximize optical transparency for “invisible” wearable biomedical sensors which adds design constraints. It should be noted that as with strain sensors for motion detection, most of the research focus for respiration detection is on maximizing strain sensitivity. Again, although sensitivity is a significant factor in accurate detection, signal resolution and sensor hysteresis may also have substantial contributions to sensor performance. And while the work listed here demonstrates that these strain sensors are capable of detecting respiration, only one work has conducted clinical correlation studies for respiration (Chu et al.). Clinical validation studies must be done in future work for practical use as healthcare monitoring devices.

### 4.3. Consumer Use

**Figure 21:**
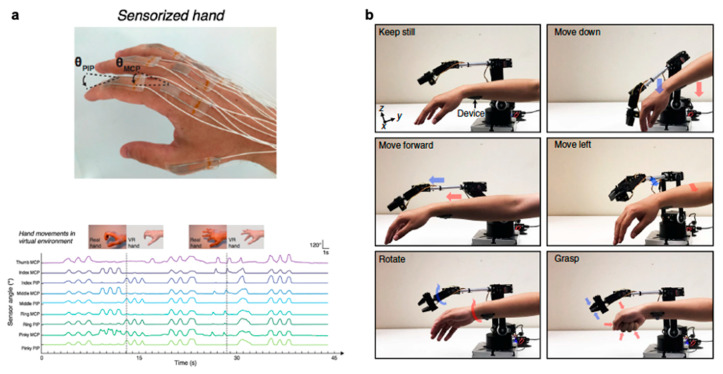
(**a**) Hand movement tracking for virtual reality applications [252]. Adapted and reproduced with permission as licensed under the Creative Commons Attribution 4.0 International License. (**b**) Wireless robotic arm control with electromyograph (EMG) data acquired from various arm motions. Reproduced with permission from [24]. Copyright 2017, Springer Nature.

**Figure 22:**
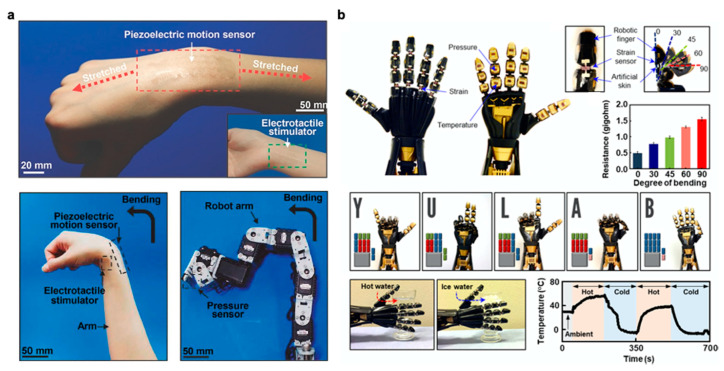
(**a**) Human motion to control a robot arm with corresponding position of the robot arm. Reproduced with permission from [253]. Copyright 2017, John Wiley and Sons (**b**) Demonstration of an intrinsically stretchable rubbery electronics-based robotic skin [254]. Adapted and reproduced with permission as licensed under the Creative Commons Attribution 4.0 International License.

Stretchable soft sensors also have a large potential role in smart human-machine interfaces with smart gloves or gesture-controlled robots. Interfacing soft mechanical sensors for virtual reality and interactive gaming to allow a subject to control a virtual environment [24,252] (as demonstrated in Figure 21) would have advantages over optical motion capture systems in terms of mobility, resolution, and cost with image processing and camera requirements. Additionally, researchers take composite approaches to create electronic skins that are capable of detecting multiple stimuli across various modalities and able to provide tactile sensing and haptic feedback, expanding on the field of soft robotics. For example, Lim et al. created an interactive human-machine interface system that combines a piezoelectric motion sensor and electrotactile simulator mounted onto the wrist to then control a robot arm through human bending motion [253]. The motion sensor is a composite sensor composed of a polylactic acid (PLA), a piezoelectric polymer, and single wall CNTs (which improve the piezoelectric power generation performance) layer that is sandwiched between graphene electrodes that are then insulated with deformable polymethyl methacrylate (PMMA). The electrotactile simulator, which is composed of AgNWs sandwiched between graphene layers and supported on PDMS, relays information to a piezoelectric pressure sensor mounted onto a robot arm (as shown in Figure 22a). Another composite electronic skin introduced by Kim et al. also incorporates multiple types of conductive fillers within an elastomer to bridge potential conductive gaps [254]. Specifically, poly(3-hexylthiophene-3,5-diyl) nanofibrils (P3HT-NFs), a conjugated semiconductor polymer, and gold nanoparticles with conformally coated silver nanowires (AuNP-AgNW) are dispersed within PDMS to create composite stretchable electronic materials. The resulting strain, pressure, and temperature sensors are able to withstand 50% strain, 1.2 MPa of pressure, and temperature to 50 °C and can be constructed into smart artificial skins for robot hands, as shown in Figure 22b.

**Figure 23:**
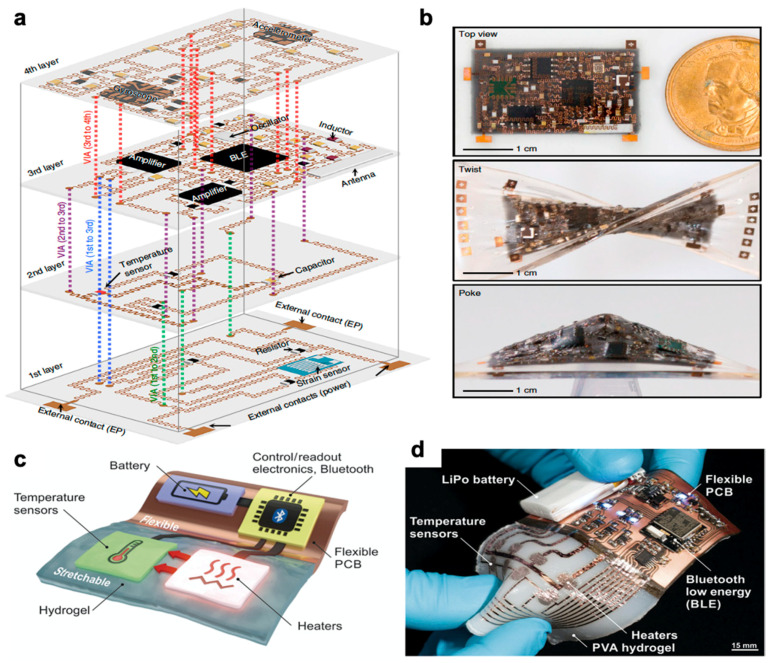
(**a**) Exploded schematic of a four-layer electronic system and (**b**) demonstration of the system’s ability to withstand mechanical deformation. Reproduced with permission from [24]. Copyright 2019, Springer Nature. (**c**) Concept and (**d**) photograph of hydrogel electronic skin [64]. Adapted and reproduced with permission as licensed under the Creative Commons Attribution 4.0 International License.

Other sensor-integrated platforms for multifunctional capabilities are examples of human-machine interfaces outside of soft robotics. Huang et al. introduce 3D-integrated stretchable electronic systems with interlayer electrical connectivity enabled through laser ablation and controlled soldering [24]. The device is built layer-by-layer (Figure 23a) and relies on a structural island-bridge mechanism to offer mechanical compliance with the islands operating as functional components and the bridge constructed of copper/polyimide (Cu/PI) serpentine-patterned thin films that are able to buckle under mechanical deformation (as demonstrated in Figure 23b). Also, typically, the adhesion of hydrogels to other materials still proves challenging for fabrication platforms, but Wirthl et al. have managed to resolve this adhesion issue with a bonding agent of cyanoacrylates diluted in alkanes to create a hydrogel electronic skin with an island-bridge construct (Figure 23c,d) [64].

Additionally, microfabrication is a key aspect in creation of high-density and multifunctional devices such as these. Without the ability to pattern with high-resolution, crafting these high-fidelity structures would be difficult to achieve. Section 2.2 briefly covered the types of traditional conductive materials that have been reduced from bulk level to nanoscale materials; we have reached a point where engineering thin films has become practically common place in the stretchable electronics field. Again, Rogers’ group has made noteworthy contribution with their ultra-thin, high resolution, conformal sensors and circuitry [19,72,73,255]. Advances in photolithography and soft lithography have allowed for micron and nanoscale resolution in transfer printing to soft polymeric materials [256] along with self-assembled microfabrication, inkjet printing, and 3D printing of soft materials [29,257,258,259,260].

**Figure 24:**
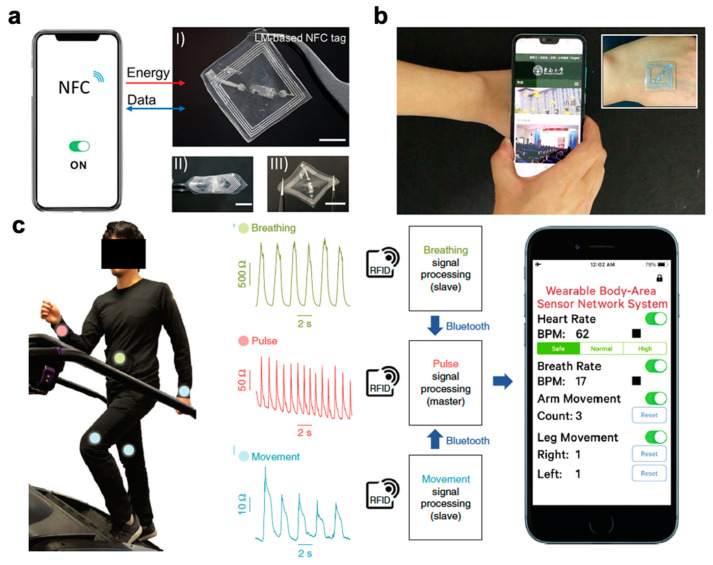
(**a**) Liquid metal-based hydrogel NFC tag with demonstrated use (**b**). Reproduced with permission from [111]. Copyright 2020 John Wiley and Sons. (**c**) Demonstration of bodyNET system with corresponding signal outputs for breathing, pulse, and arm movement. Reprinted with permission from [238]. Copyright 2019, Springer Nature.

There have also been recent developments in stretchable on-skin tags what would wirelessly transmit human physiological signals, that could have promising application in healthcare monitoring, athletic performance, and entertainment. Niu et al. developed a body area sensor network (bodyNET) (demonstrated in Figure 24c) with a collection of SEBS-based passive tags using silver conductive ink [238]. These sensors were then mounted onto the body using Tegaderm, a medical-grade adhesive. Other groups created hydrogel-based wireless antennas. Lim et al. fabricate a wireless antenna with a nanocomposite AgNW/alginate hydrogel supported by a PAAm-hydrogel substrate [261] whereas Xu et al. display high-resolution patterning of liquid metal on a PVA hydrogel for near-field communication (NFC) (Figure 24a,b) [111].

## 5. Challenges and Future Outlook

This review aims to provide an overview of materials advances, particularly in polymer materials, made in soft stretchable electronics to date and to review their behavior under mechanical deformation. We covered materials considerations, sensor performance metrics, and applications. Materials innovations have involved both creating novel polymers for stretchable electronics using sophisticated molecular design and synthesis as well as hybrid material combinations of existing materials for novel device performance. Progress in hydrogels and supramolecular polymers have allowed various novel properties such as self-healing and self-adhesion. Despite these advances, stretchable electronics composed of these new materials also remain less conductive than traditional conductors. Advances in soft materials and chemistry will be essential to future progress in the field of stretchable electronics with an expansion on the existing library of soft stretchable materials for active conductive elements, polymer matrices and encapsulation, and power supply. In addition to raising the conductivity in intrinsically stretchable conductors, future work on mechanical properties of wearable polymer materials need to include physical robustness and durability for protection from handling along with potential of long-duration wear times and take into account the cycles of application and removal. Other integration challenges for skin-interfaced stretchable systems also include non-idealized conditions (e.g., sweat, hair follicles, dynamic nonuniform skin conditions) and dynamic environmental factors influencing sensing accuracy. Moreover, a robust device/skin interface will be an important aspect where a soft, biocompatible material is desired for conformal contact with the skin and also one that allows for a breathable interface for long-term use.

While there have been many advances in fabrication of and materials considerations for soft stretchable sensors, challenges remain in translating research of stretchable conductors to commercialization with scale and manufacturing. Progress will be dependent on integration of soft materials molding techniques, roll-to-roll and lamination processes, pick and place assembly protocols, and biocompatible skin adhesive interfaces to allow for advanced manufacturing in soft electronics. As evident of the multifunctional platforms briefly covered in Section 4.3, strategies will need to be developed to create fully integrated devices of increasing complexity. They need to take into account the number of assembly operations, heterogeneity and spatial distribution of the multimodal sensors, power requirements, and operation lifetimes. Examples of commercial skin-interfaced wearable systems in the market include StretchSense silicone stretch sensors [262], Vital Connect VitalPatch for hospital patience health monitoring [263] GE healthcare Novii Wireless Patch & Pod System for fetal monitoring [264], mc10 Biostamp nPoint wearable sensing patch [265], and PyrAmes Health continuous non-invasive blood pressure monitoring system [266]. VitalPatch, Novii, and Biostamp represent FDA 510(k) cleared wearable medical devices with multimodal data, wireless connectivity, and conformal electromechanical structures. While this list represents a few commercially available cases, system level challenges remain with seamless integration of sensors, power supplies, and wired/wireless communication connections. Further, limitations in power supply are an active research area with new requirements in form factor, size, and weight along with rising demands in computational power, communication bandwidth, operating distances, and operation lifetimes.

Overall, the state of technology with soft materials and stretchable wearable systems will require verification testing, validation studies, and cost-effective manufacturing to enable widescale adoption. There must also be long-term operation stability with human factors in device attachment, removal, placement, recharging, and disposal placing other constraints on materials choices and design. Future systems will need manufacturing efficiency, reliability and calibration testing, appropriately tailored electromechanical properties, low power requirements, insulation from signal noise and other outside environmental influence, and still remain breathable to allow for passage of sweat and other necessary biofluids. Research in this field will be a highly interdisciplinary effort with the technical challenges spanning a wide number of disciplines in engineering and materials science and medical science.

## Figures and Tables

**Table 1:** Mechanical properties of common elastomers (from their technical data sheets).

Elastomer	Commercial Name	Material Type	Young’s Modulus [MPa]	Elongation at Break [%]
Poly(dimethylsiloxane)	Sylgard-184	Silicone	0.4–3.5	80–170%
Silicone Elastomer	Ecoflex-30	Silicone	0.45–0.69	800–1000%
Silicone Elastomer	Dragon Skin	Silicone	0.15–0.6	364–1000%
Polyurethane	Elastollan	Thermoplastic	1.7–13.8	400–720%
Styrene-butadiene-styrene (SBS)	Kraton D	Thermoplastic	1.2–2.9	600–880%
Styrene-ethylene-butadiene-styrene (SEBS)	Kraton G	Thermoplastic	2.9–5.5	600–1200%

**Table 2:** Summary of performances of representative wearable strain sensors for motion detection reported.

Materials	Conductive Type	Sensing Type	GF	Sensing Range	Application	Ref.
Silver nanofibers/ionic hydrogel	Ionic	Capacitive	165	1000%	Joint motion, physiological, facial expression	[223]
Conductive tape(eCAP)/PDMS	Electronic	Capacitive	0.9	150%	Gesture Detection	[224]
Ultrathin wrinkled gold/Parylene	Electronic	Capacitive	3.05	140%	Finger joint bending	[169]
AgNWs /Ecoflex/PDMS	Electronic	Capacitive	0.7	50%	Joint bending, motion detection (walking, running, squatting, jumping)	[83]
Al/Ag/Dragon Skin	Electronic	Capacitive	0.9	250%	Elbow joint bending	[225]
Silver plated knitted textile/Ecoflex	Electronic	Capacitive	1.23	150%	Hand motion tracking	[226]
PU-PEDOT:PSS/ single wall CNTs/PU-PEDOT: PSS	Ionic/Electronic	Resistive	62	100%	Facial expressions, eye movement	[3]
AgNWs /PDMS	Electronic	Resistive	84	40%	Swallowing, finger/knee bending,	[14]
Cracked Platinum/PU	Electronic	Resistive	30	150%	Whole body motion	[162]
Silver nanoparticles/PDMS	Electronic	Resistive	2.05	20%	Joint bending	[219]
CNTs /Ecoflex	Electronic	Resistive	256 (0–80%), 3250 (80–25%), 42,300 (125–145%)	145%	Joint bending, swallowing, vocal phonation	[221]
AgNW microgrids/PDMS	Electronic	Resistive	6.9 (0–30%), 41.1 (30–35%)	35%	Joint bending, eye movement, throat movement,	[81]
Microstructured AgNWs/PDMS	Electronic	Resistive	81	150%	Joint bending, throat movement, eye movement, respiration, wrist flexion	[227]
Laser-induced graphene/Ecoflex	Electronic	Resistive	457 (30%), 268 (100%)	100%	Finger pulse, respiration, vocal phonation	[228]
Graphene foam/PDMS	Electronic	Resistive	24	70%	Neck posture, radial and brachial pulse detection	[229]
PVA/PDA hydrogel	Ionic	Resistive	-	500%	Facial expressions, joint bending, respiration, throat movement	[230]
Zinc oxide nanorods/PDMS/AgNWs/single walled CNTs	Electronic	Piezoelectric	-	-	Finger bending	[231]
PANI/PAA/PA	Electronic	Resistive	11.6 (100%), 4.7 (>100%)	425%	Joint motion, hand fist movement, vocal phonation, respiration	[145]
PVDF nano/microfibers/PDMS/Ecoflex	Electronic	Piezoelectric	-	>300%	Wrist flexion, respiration, walking motion	[232]
Carbon nanofibers/PU	Electronic	Resistive	72	300%	Joint motion	[233]
Polypyrrole/TPU	Ionic/Electronic	Resistive	-	14,500%	Posture detection, walking motion, fall detection	[148]
eGaIn alloy/Ecoflex	Electronic	Resistive	2.2–2.5	400%	Body motion, gait measurement	[182]
GaInSn/PDMS	Electronic	Resistive	2	50%	Wrist flexion, vocal cord movement, finger motion	[112]
Gold nanowire-based film/Ecoflex	Electronic	Capacitive or Resistive	-	900%	Facial expression detection	[4]
Multiwalled CNTs/TPU	Electronic	Resistive	2800 (5–100%)	120%	Joint motion	[99]
Potassium Iodide/Glycerol/Ecoflex	Ionic	Resistive	2.2 (50%)	-	Index finger bending	[234]
